# Supplementary material for: Stabilization of DNA fork junctions by Smc5/6 complexes revealed by single-molecule imaging
Source: Cell Rep. 2022 Dec 6;41(10):111778. doi: 10.1016/j.celrep.2022.111778 (PMC9756111; doi:10.1016/j.celrep.2022.111778)
Supplement: Document S1. Figures S1–S6 and Table S1 [file mmc1.pdf]

Cell Reports, Volume *41*

Supplemental information

Stabilization of DNA fork junctions by Smc5/6 complexes revealed by single-molecule imaging

Nicoleta-Loredana Tanasie, Pilar Gutiérrez-Escribano, Sigrun Jaklin, Luis Aragon, and Johannes Stigler

Supplemental information

Supplemental figures

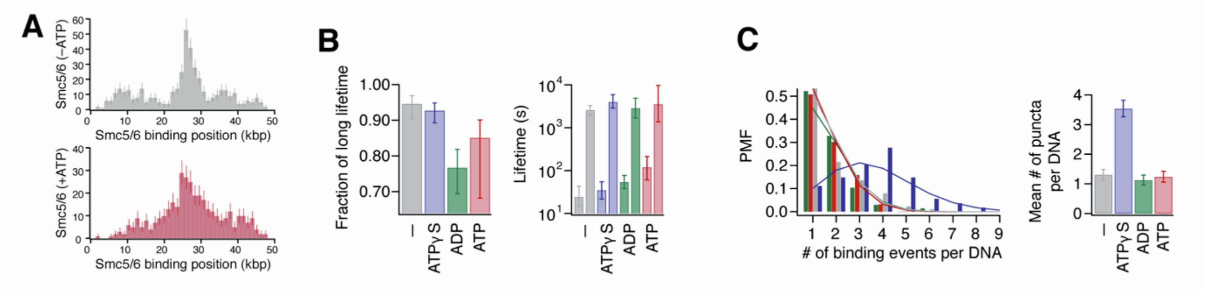

Figure S1. Nucleotides regulate the Smc5/6-DNA interaction, Related to **Fig. 1**. **(A)** Histograms of Smc5/6 binding locations in the absence of nucleotide (*n*=441, top) or in the presence of ATP (*n*= 366, bottom). **(B)** Fraction of long-lived population (left) and lifetimes of short-lived and long-lived populations (right) from double exponential fits to survival curves. Grey: no nucleotide, blue: ATPγS, green: ADP, red: ATP. **(C)** Left: distributions of the number Smc5/6 binding events per DNA, depending on nucleotide. Lines are fits to a Poissonian distribution model. Right: mean number of puncta per DNA depending on nucleotide. Color code as in (B). Data were collected from at least 50 DNA molecules per nucleotide condition. All error bars represent 68% CIs from bootstrapping.

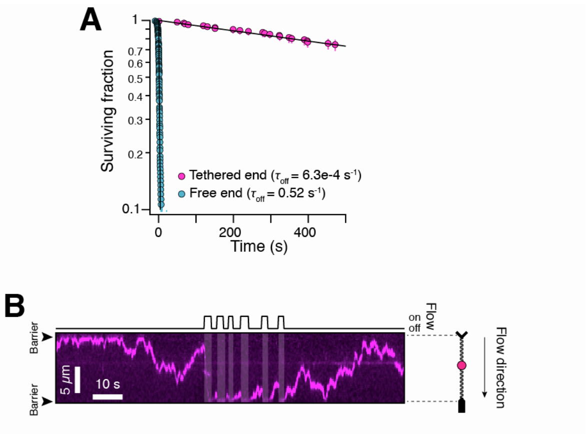

Figure S2. Smc5/6 interacts topologically with DNA, Related to **Fig. 2**. **(A)** Survival plots of moving complexes on DNA after an injection of 250 mM NaCl, on topologically unconstrained single-tethered DNA (*n*=180, cyan) and topologically constrained double-tethered DNA (*n*=97, magenta). Data were collected from at least 50 DNA molecules. **(B)** Smc5/6 movement on double-tethered DNA at 250 mM NaCl. Buffer flow pushes the complex toward the lower barrier.

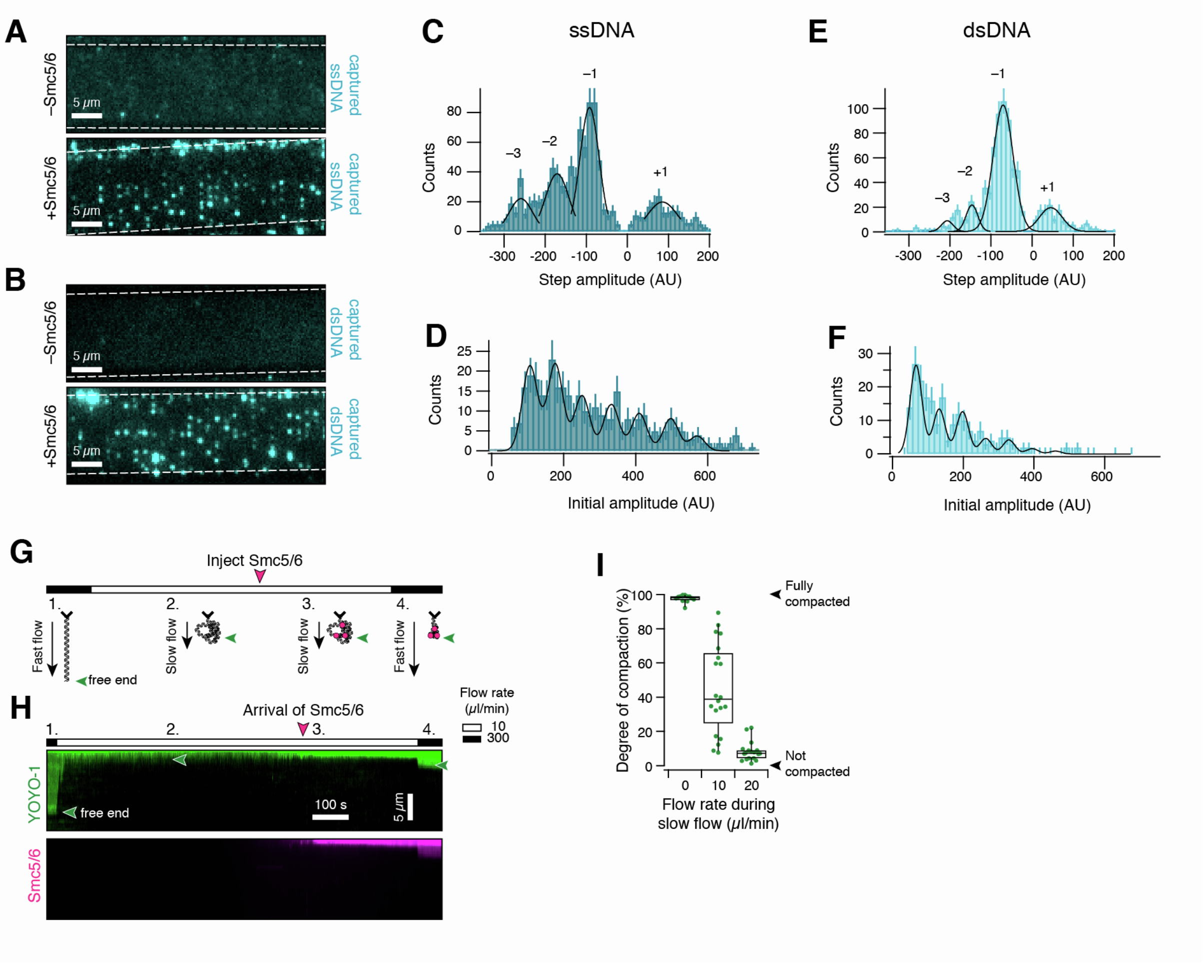

Figure S3. Smc5/6 can capture multiple ss or dsDNA oligos and compact dsDNA, Related to **Fig. 3**. **(A)** Wide-field images of dsDNA curtains with ssDNA-Alexa594 in solution. Top: without previous loading of Smc5/6 onto the curtain. Bottom: with previous loading of Smc5/6 onto the curtain. **(B)** Wide-field images of dsDNA curtains with dsDNA-Alexa594 in solution. Top: without previous loading of Smc5/6 onto the curtain. Bottom: with previous loading of Smc5/6 onto the curtain. **(C)** Detected step size histogram of photobleaching trajectories of captured ssDNA-Alexa594 oligos after flush-out. Black lines indicate the bleaching of a single fluorophore (–1), two fluorophores (–2), etc. Because the oligo was not completely removed from the flow cell, there are also occasional re-capture events (+1), seen by an increase in the fluorescence amplitude. **(D)** Histogram of the initial amplitude of captured ssDNA puncta after flush-out (*n*=387). Black lines represent a fit to the histogram with multiple gaussians corresponding to the fluorescence of one, two, etc. captured oligos. **(E,F)** see (C,D), but for the capture of dsDNA-Alexa594 (*n*=222). Histograms data were collected from at least 40 DNA molecules. Error bars are 68% confidence intervals. **(G)** Experimental profile of a flow-stretch compaction assay. **(H)** Representative kymogram of YOYO-1-stained DNA during compaction by Smc5/6 under buffer flow. Green arrowheads indicate the free end position. **(I)** The degree of compaction depends

on the buffer flow rate during the incubation phase. High flow, *i.e.* stretched DNA, prevents compaction. Data were collected from at least 12 DNA molecules per condition and are represented as box plots showing the median ± quartiles with whiskers indicating 9th and 91st percentiles.

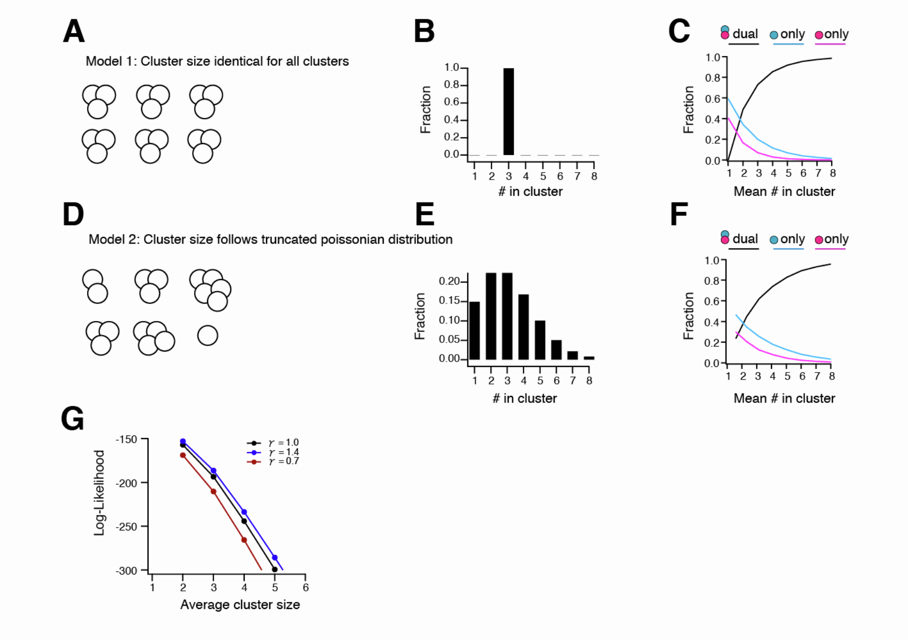

Figure S4. Models for determining the degree of oligomerization, Related to **Fig. 4**. **(A)** Cluster size model 1: All detected puncta have the same oligomeric size. **(B)** Distribution of oligomeric state according to model 1. **(C)** Prediction of the fraction of cyan-only-labeled, magenta-only-labeled and dual-color-labeled puncta according to model 1. **(D)** Cluster size model 2: The distribution of the number of molecules per punctum follows a truncated Poissonian distribution. **(E)** Assumed distribution according to model 2. **(F)** Predicted fraction of single and dual colored puncta according to model 2. **(G)** The likelihood function and its prediction of cluster size is largely unaffected by differences in the ratio γ of the labeling efficiency of cyan vs magenta labels.

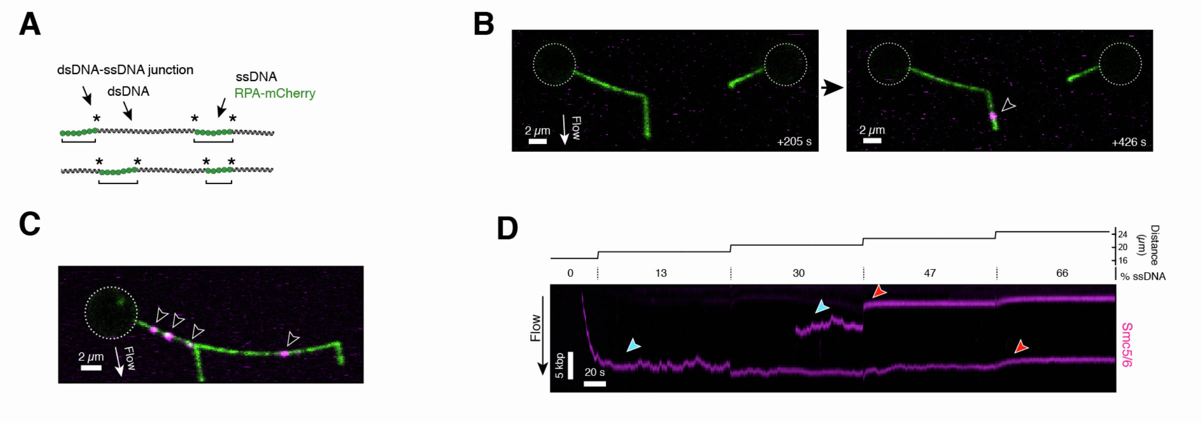

Figure S5. Smc5/6 interacts with ssDNA-dsDNA hybrids, Related to **Fig. 5**. **(A)** Schematic depiction of how the fractions of available dsDNA and ssDNA are determined (see **STAR Methods**). Asterisks denote junctions. **(B)** Perpendicular flow images of ssDNA/RPA-dsDNA hybrids. Green: RPA, magenta: Smc5/6. In this example, Smc5/6 binds directly to the unpeeled ssDNA strand (arrowhead). **(C)** Example where Smc5/6 complexes bind directly to the tethered ssDNA. **(D)** Kymogram of Smc5/6 movement on DNA during stepwise melting of the DNA. Blue arrows highlight mobile complexes. Higher trap distances, *i.e.* higher forces, result in local melting and increased exposure of ssDNA. Even in the absence of RPA, diffusing Smc5/6 complexes suddenly stop moving, presumably when they encounter ssDNA (red arrowheads).

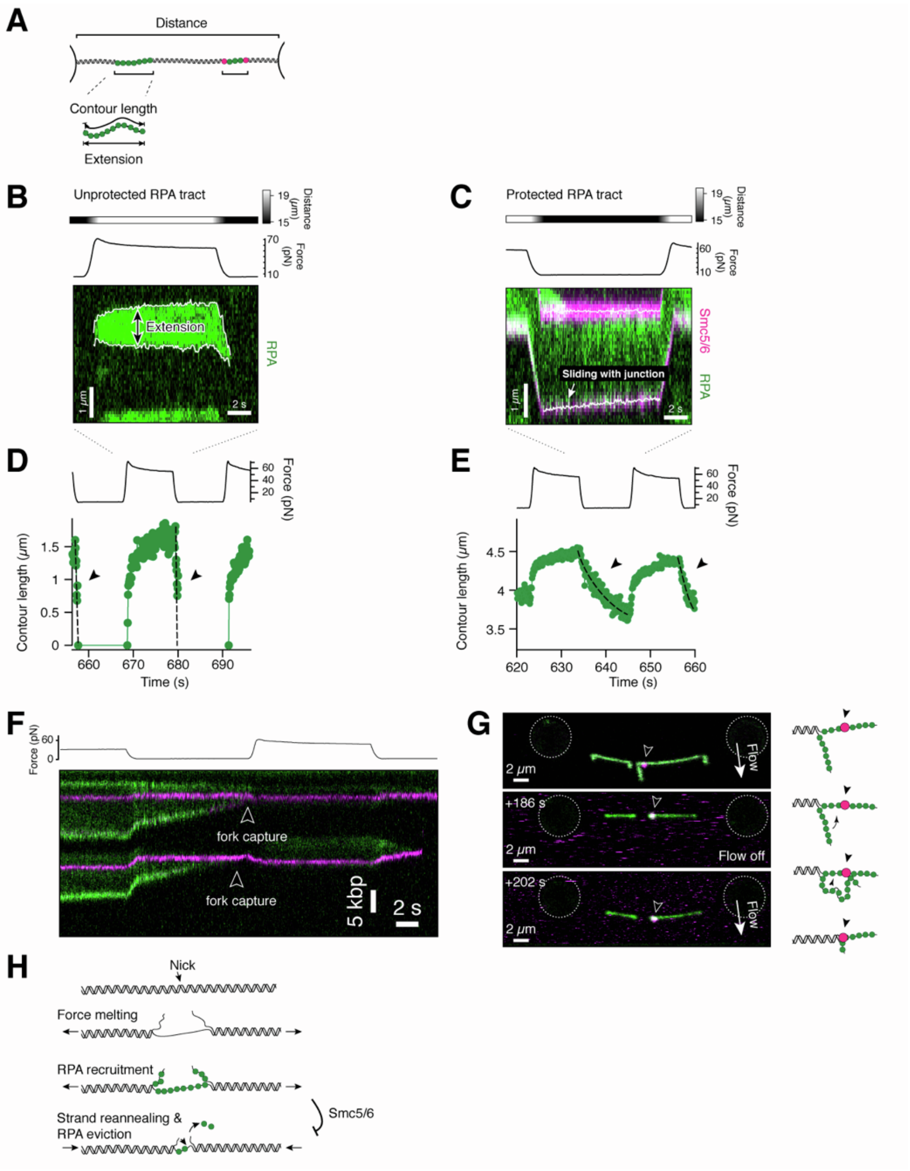

Figure S6. Smc5/6 associates with fork junctions, Related to **Fig. 6**. **(A)** The experimentally visible extensions of ssDNA tracts and dsDNA tracts are affected differently under force manipulation because ssDNA, even if it is RPA covered, has a much lower persistence length than dsDNA. The extension of tracts, which is directly experimentally accessible, is hence not a good reporter of the true tract length, *i.e.* the contour length. The shown schematic highlights this difference. **(B)** Kymogram of the extension of an unprotected RPA tract during force manipulation. White lines represent the RPA tract boundaries from tracking. **(C)** Kymogram of the extension of a protected RPA tract, *i.e.* with Smc5/6 bound to both tract junctions, during force manipulation. White lines represent the tract boundaries from tracking. **(D)** Contour length timeline of an unprotected RPA tract during force manipulation. The tract size rapidly returns to zero at low force (arrowheads). **(E)** Contour length timeline of a Smc5/6-protected RPA tract during force manipulation. The protected tract is kept open by Smc5/6. Nevertheless, even the protected tract shows slow dynamics due to sliding junctions (arrowheads). **(F)** Example kymogram showing that Smc5/6 bound to ssDNA associates with the ssDNA-dsDNA fork upon encounter (also see **Fig. 6E**). **(G)** Example video of

Smc5/6 (magenta) binding to the tethered ssDNA under perpendicular flow, followed by junction recruitment. At reduced force and in the absence of flow, the unpeeled ssDNA reanneals and the RPA to the left of the Smc5/6 complex is evicted. When the perpendicular flow is reinstated, Smc5/6 is at the junction and has possibly compacted part of the ssDNA. **(H)** Cartoon interpretation of the data in **Fig. 6**. An increase in force exposes ssDNA regions around nicks and lesions which become covered by RPA. In the absence of Smc5/6, reannealing of the displaced strand evicts RPA. This eviction is counteracted by the stabilization of ssDNA-dsDNA fork junctions by Smc5/6.

Supplemental tables

Table S1. Mass spectrometry verification of Smc5/6 holocomplexes, Related to **Fig. 1**. SM: Number of significant matches. SS: Number of significant sequences. emPAI: Exponentially Modified Protein Abundance Index

Accession	Score	Mass	SM	SS	emPAI	Description
**SMC6_YEAST**	27148	128568	784	73	9.24	Structural maintenance of chromosomes protein 6 OS=Saccharomyces cerevisiae (strain ATCC 204508 / S288c) OX=559292 GN=SMC6 PE=1 SV=1
**SMC5_YEAST**	10975	126481	408	75	9.97	Structural maintenance of chromosomes protein 5 OS=Saccharomyces cerevisiae (strain ATCC 204508 / S288c) OX=559292 GN=SMC5 PE=1 SV=1
**RLA0_YEAST**	104	33696	5	4	0.58	60S acidic ribosomal protein P0 OS=Saccharomyces cerevisiae (strain ATCC 204508 / S288c) OX=559292 GN=RPP0 PE=1 SV=2
**NSE5_YEAST**	15312	64784	511	22	2.71	Non-structural maintenance of chromosome element 5 OS=Saccharomyces cerevisiae (strain ATCC 204508 / S288c) OX=559292 GN=NSE5 PE=1 SV=1
**NSE4_YEAST**	2382	46150	93	19	3.88	Non-structural maintenance of chromosome element 4 OS=Saccharomyces cerevisiae (strain ATCC 204508 / S288c) OX=559292 GN=NSE4 PE=1 SV=1
**NSE3_YEAST**	6521	34002	265	20	9.77	Non-structural maintenance of chromosome element 3 OS=Saccharomyces cerevisiae (strain ATCC 204508 / S288c) OX=559292 GN=NSE3 PE=1 SV=1
**NSE2_YEAST**	1288	30857	53	9	2.06	E3 SUMO-protein ligase MMS21 OS=Saccharomyces cerevisiae (strain ATCC 204508 / S288c) OX=559292 GN=MMS21 PE=1 SV=2
**NSE1_YEAST**	6810	38890	235	11	1.98	Non-structural maintenance of chromosomes element 1 OS=Saccharomyces cerevisiae (strain ATCC 204508 / S288c) OX=559292 GN=NSE1 PE=1 SV=1
**KRE29_YEAST**	6637	54260	303	27	5.82	DNA repair protein KRE29 OS=Saccharomyces cerevisiae (strain ATCC 204508 / S288c) OX=559292 GN=KRE29 PE=1 SV=1
