# Supplementary material for: Stabilization of DNA fork junctions by Smc5/6 complexes revealed by single-molecule imaging
Source: Cell Rep. 2022 Dec 6;41(10):111778. doi: 10.1016/j.celrep.2022.111778 (PMC9756111; doi:10.1016/j.celrep.2022.111778)
Supplement: Document S2. Article plus supplemental information [file mmc2.pdf]

Cell Reports

Article

Stabilization of DNA fork junctions by Smc5/6 complexes revealed by single-molecule imaging

Graphical abstract

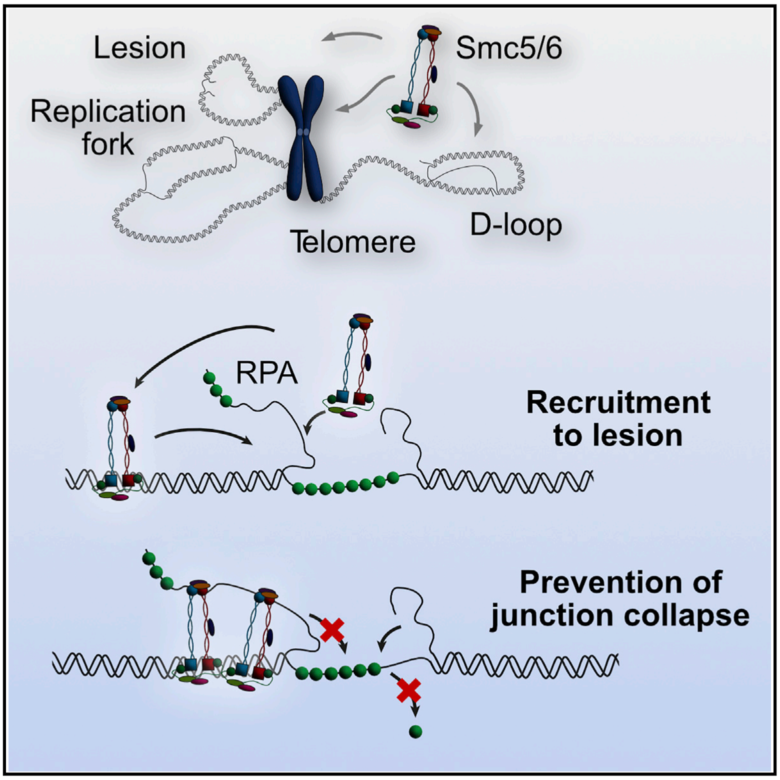

Highlights

- The Smc5/6 holocomplex can form oligomers and translocate on double-stranded DNA
- The complex can simultaneously bind single-stranded and double-stranded DNA
- Smc5/6 is recruited to ssDNA-dsDNA junction structures mimicking replication forks
- The holocomplex indirectly stabilizes the ssDNA-binding protein RPA on ssDNA

Authors

Nicoleta-Loredana Tanasie,
Pilar Gutiérrez-Escribano, Sigrun Jaklin,
Luis Aragon, Johannes Stigler

Correspondence

stigler@genzentrum.lmu.de

In brief

Tanasie et al. combine single-molecule microscopy and force manipulation to investigate the interaction of the Smc5/6 holocomplexes with fork junction structures mimicking DNA replication and repair intermediates. They show that Smc5/6 targets and stabilizes these DNA substrates, suggesting a model for the Smc5/6-mediated recognition of replication stress or DNA lesions.

Tanasie et al., 2022, Cell Reports *41*, 111778
December 6, 2022 © 2022 The Authors.
https://doi.org/10.1016/j.celrep.2022.111778

CellPress

CellPress
OPEN ACCESS

Article

Stabilization of DNA fork junctions by Smc5/6 complexes revealed by single-molecule imaging

Nicoleta-Loredana Tanasie,[1] Pilar Gutiérrez-Escribano,[2] Sigrun Jaklin,[1] Luis Aragon,[2] and Johannes Stigler[1,3,*]
[1]Gene Center, Ludwig-Maximilians-University, 81377 Munich, Germany
[2]DNA Motors Group, Medical Research Council London Institute of Medical Sciences, Du Cane Road, London W12 0NN, UK
[3]Lead contact
*Correspondence: stigler@genzentrum.lmu.de
https://doi.org/10.1016/j.celrep.2022.111778

SUMMARY

SMC complexes play key roles in genome maintenance, where they ensure efficient genome replication and segregation. The SMC complex Smc5/6 is a crucial player in DNA replication and repair, yet many molecular features that determine its roles are unclear. Here, we use single-molecule microscopy to investigate Smc5/6's interaction with DNA. We find that Smc5/6 forms oligomers that dynamically redistribute on dsDNA by 1D diffusion and statically bind to ssDNA. Using combined force manipulation and single-molecule microscopy, we generate ssDNA-dsDNA junctions that mimic structures present in DNA repair intermediates or replication forks. We show that Smc5/6 accumulates at these junction sites, stabilizes the fork, and promotes the retention of RPA. Our observations provide a model for the complex's enrichment at sites of replication stress and DNA lesions from where it coordinates the recruitment and activation of downstream repair proteins.

INTRODUCTION

Chromosomal breaks are a considerable threat to the viability of cells. Unrepaired, these lesions may lead to the loss of genetic information or cell death. Hence, DNA repair is essential for proper cell proliferation. Smc5/6, a member of the structural maintenance of chromosomes (SMC) complexes, has been extensively associated with various DNA repair processes.[1] SMC complexes as a group regulate chromosome structure and maintain genome stability by ensuring efficient genome replication and segregation, each member sharing a similar architecture and contributing to specific genome maintenance processes.[2,3]

At its core, Smc5/6 consists of two SMC subunits (Smc5 and Smc6) that feature anti-parallel coiled coils that dimerize at the hinge domain and connect to an ATPase domain at the head region. The Smc5/6 dimer further associates to six non-SMC elements (Nses), referred to as Nse1 through Nse6 in yeast.[1] The complex was first identified in genetic screens against radiation-induced DNA damage[4] and later associated with double-strand break (DSB) repair via homologous recombination (HR),[5–7] an error-free repair pathway that uses the sister chromosome as a repair template.

DNA repair is closely intertwined with DNA replication to promote genome stability.[8] Besides repetitive genomic regions, DNA lesions are a major hurdle encountered by the DNA replication machinery and can lead to stalling or even collapse of the replication fork.

Homologous recombination promotes the restart of the replication fork in a pathway involving DSBs. DSB repair by HR depends on the single-stranded DNA (ssDNA) binding protein RPA (replication protein A) as a precursor for the assembly of Rad51 recombinase filaments that invade the homologous chromosome copy to form a ssDNA-dsDNA D-loop repair intermediate. This is later on resolved by synthesis-dependent strand annealing or the formation and subsequent resolution of a double-Holliday junction in DSB repair.[9–11]

Smc5/6 is associated with several processes in the context of challenged replication forks.[12] The complex has an early role in replication, where it localizes to stalled replication forks and maintains them in a stable restart-prone conformation by promoting recruitment of RPA and Rad52.[13] Furthermore, Smc5/6 interacts with the Mph1 helicase at damaged forks to promote fork regression and prevent error-prone fork restart.[14] HR can lead to recombination intermediates that need to be removed before chromosome segregation to avoid the accumulation of concatenated joint molecules and toxic stress. Smc5/6 plays a role in resolving recombination intermediates, where it recruits the STR (Sgs1-Top3-Rmi1) complex to DNA damage sites through interaction between the SUMOylated Smc5/6 complex and the SUMO-interacting motifs (SIMs) of Sgs1.[15,16]

Replication stress is especially prevalent in repetitive telomeric regions, where the failure to recover from stalled replication forks leads to senescence.[17] At these sites, Smc5/6 is involved in the alternative lengthening of telomeres (ALT) maintenance pathway, by facilitating HR through SUMOylation of telomere binding proteins.[18]

Proper removal of recombination intermediates is particularly important in meiosis, where recombination at programmed DSBs leads to crossovers between parental genomes.[19] Failure to resolve these intermediates can lead to blockage of

© 2022 The Authors.
This is an open access article under the CC BY license (http://creativecommons.org/licenses/by/4.0/).

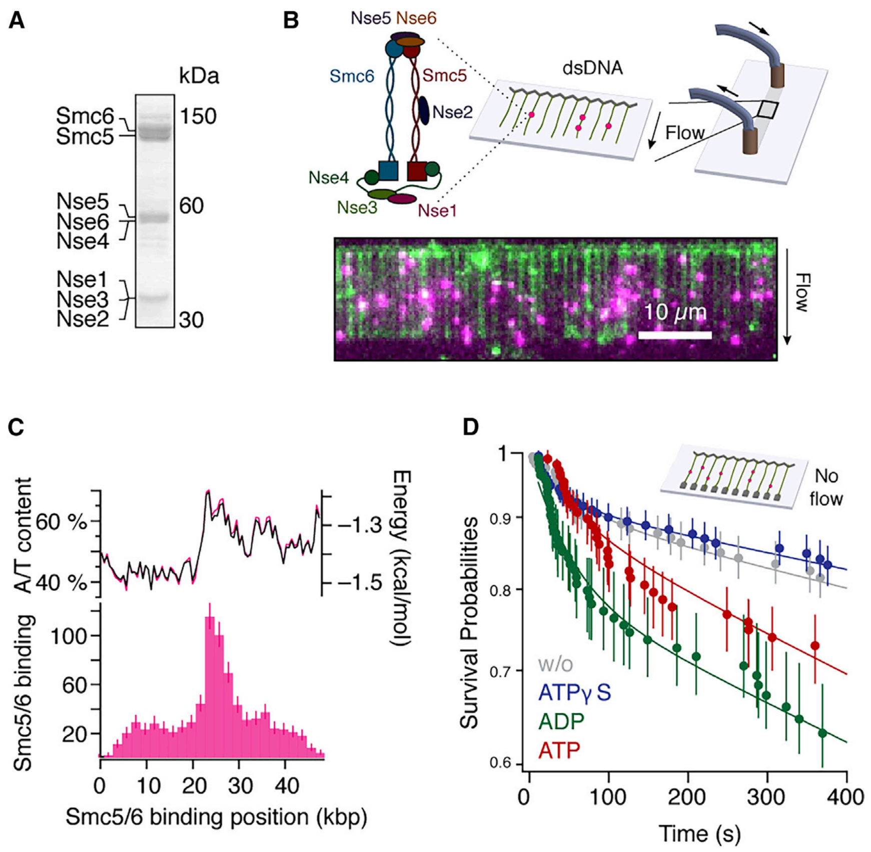

Figure 1. Recombinant Smc5/6 holocomplex interacts with DNA in a nucleotide-dependent manner

(A) Coomassie-stained PAGE gel of purified Smc5/6 holocomplex.

(B) Single-tethered DNA curtains assay to study the binding location of Qdot-tagged Smc5/6 (magenta) on flow-stretched, YOYO-1-stained λ-DNA (green).

(C) Histogram of recorded Smc5/6 binding location on λ-DNA. Top: A/T content (black) and base-pairing energy[24] (magenta) of the DNA substrate (n = 807).

(D) Survival curves of Smc5/6 on dsDNA in the absence of flow under different nucleotide conditions. Gray, no nucleotide (n = 166); blue, ATPγS (n = 194); green, ADP (n = 114); red, ATP (n = 108). Data were collected from at least 50 DNA molecules per condition. Continuous lines are fits to a double exponential model. Error bars represent 68% confidence intervals. See also Figure S1 and Table S1.

chromosome segregation.[20] Interestingly, Smc5/6 is specifically recruited to meiotic DSBs to promote the removal of inappropriate joint molecules.[20]

Smc5/6 has an established role in DNA repair. Nevertheless, the detailed function of the complex at sites of DNA damage remains unclear. As a complex involved in the maintenance of chromosomes, Smc5/6 binds DNA through multiple DNA binding domains and interacts with various DNA substrates, raising the possibility that the recognition of different DNA configurations might be a crucial factor for its involvement in DNA repair.[21] Only recently, single-molecule studies have been successfully employed for mechanistic studies of the complex. Using a magnetic tweezers assay, two independent studies[22,23] demonstrated the complex's ability to compact dsDNA under low forces and to stabilize supercoiled plectonemes, which can be induced during DNA replication and transcription, in an ATP-dependent manner, providing an insight into how Smc5/6 interacts with unusual DNA substrates. Nonetheless, mechanistic insight into the recruitment process to lesion sites or stressed replication forks, where the complex is exposed to both dsDNA and ssDNA, is still missing.

Here, we visualize the interaction of recombinant Smc5/6 holocomplexes from budding yeast[22] with relevant DNA substrates at the single-molecule level. We demonstrate that, on dsDNA, Smc5/6 exhibits one-dimensional (1D) diffusion, while association with ssDNA occurs in a static manner. We use a force manipulation assay to generate substrates featuring ssDNA-dsDNA junctions that mimic the structure of stalled replication forks, DNA repair intermediates or telomeres, and show that these fork junctions are targeted and stabilized by Smc5/6. In addition, we uncover that the complex dynamically oligomerizes and can capture at least one additional DNA molecule while already loaded on DNA. Our findings are consistent with predictions on Smc5/6's putative role in DNA damage repair and provide a mechanistic model for the Smc5/6-mediated recognition of lesions as a recruitment and activation platform for downstream factors.

RESULTS

Smc5/6 preferably binds to DNA regions with very high A/T content

We expressed and purified Smc5/6 holocomplexes (henceforth called Smc5/6), consisting of the two SMC subunits Smc5 and Smc6, as well as the six non-SMC subunits Nse1-Nse6 (Figure 1A), by a three-step procedure as described previously.[22] The complex purity and integrity were confirmed by gel electrophoresis and mass spectrometry (STAR Methods). The complex was fluorescently labeled using Qdots targeting a terminal HA epitope tag on the Nse4 subunit.

To assess the binding preference of Smc5/6, we first incubated fluorescently tagged Smc5/6 molecules with 48.5 kbp λ-phage DNA molecules in a low salt buffer (25 mM NaCl, 25 mM KCl) in a single-tethered double-stranded DNA (dsDNA) curtain setup, where DNA is arranged in a flow-stretched array[25] and visualized via TIRF microscopy (Figure 1B). To our surprise, unlike the related SMC complex cohesin which preferentially binds A/T rich regions,[26,27] Smc5/6 localized preferentially to the center of the λ-DNA (Figure 1C), and showed only weaker association to other sites. This binding behavior was independent of ATP presence in the flow cell (Figure S1A).

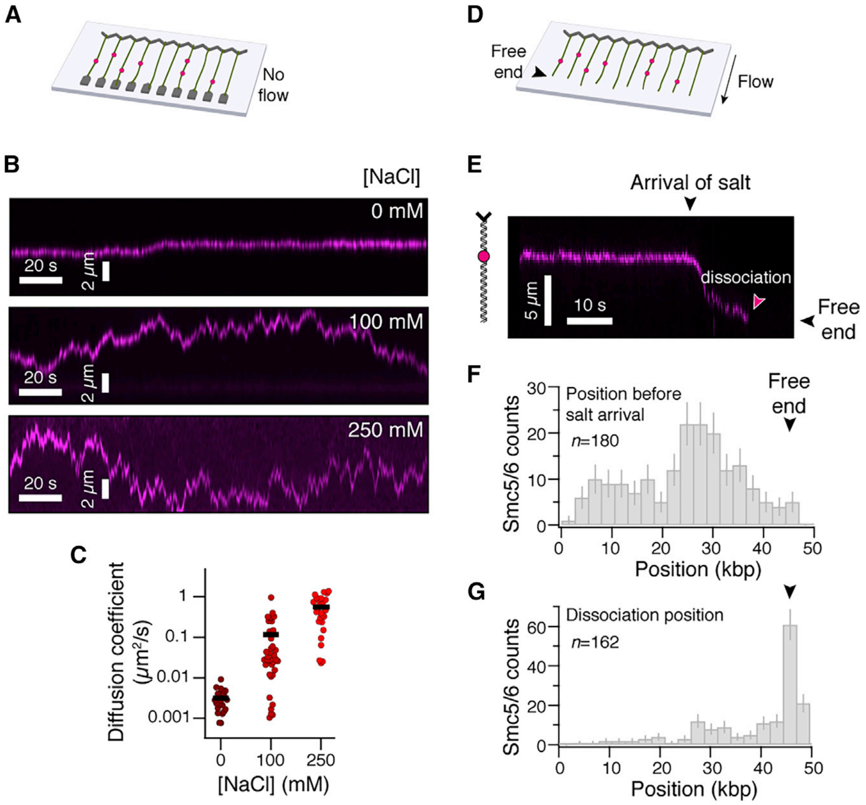

Figure 2. Salt-concentration-dependent dynamics on DNA

(A) Double-tethered DNA curtains assay to observe the movement of fluorescently tagged Smc5/6 (magenta) on DNA.

(B) Typical kymograms of Smc5/6 movement at various concentrations of NaCl.

(C) Smc5/6 diffusion coefficients at various salt concentrations: 0 mM NaCl (n = 22), 100 mM NaCl (n = 39), and 250 mM NaCl (n = 27). Black bars indicate the distribution mean.

(D) Salt-chase assay to determine the dynamics and dissociation position of Smc5/6 upon high-salt exposure.

(E) Typical kymogram of Smc5/6 behavior upon injection of 250 mM NaCl.

(F) Binding histogram before salt arrival.

(G) Histogram of dissociation positions after salt arrival. Data were collected from 104 DNA molecules. Error bars are 68% confidence intervals. See also Figure S2.

The center region of the substrate contains an exceptionally high A/T content of ~70%, which is prone to local melting,[28,29] as expected also from the base-pairing energy profile[24] for the λ-DNA (Figure 1C). We therefore speculate that Smc5/6 may recognize and be recruited to partially melted DNA structures.

ATP hydrolysis regulates the binding lifetime of Smc5/6

As Smc5/6 harbors two active ATPase sites, we wondered whether the presence of nucleotides influences the binding dynamics. To this end, we recorded the lifetimes of complexes on double-tethered λ-DNA curtains, where the free end of the λ-DNA substrate was anchored to the surface and dynamics could be observed in the absence of flow. While in the absence of nucleotide, or in the presence of the non-hydrolyzable ATP analog ATPγS, Smc5/6 exhibited extraordinarily long lifetimes with ~90% of complexes remaining bound for at least 5 min, the lifetimes became noticeably shorter in the presence of ATP or ADP (Figure 1D). Under all conditions, the binding time to the DNA can be described by two populations with different lifetimes. We quantified these by fitting a double exponential model. While there was largely no difference between ATPγS and no-nucleotide, the fraction of stable binders was lower in the presence of ATP or ADP (Figure S1B), indicating that the continuous or transient presence of ADP in the Smc5/6 binding pockets enhances Smc5/6's off-rate from the DNA. Interestingly, we also observed a significantly higher number of binding events of ATPγS-bound Smc5/6 (Figure S1C). These results indicate that the observed holocomplexes are physiologically active molecules whose DNA binding properties are influenced by the bound nucleotides.

Smc5/6 dynamically moves on dsDNA

We next sought to understand if Smc5/6 complexes can also dynamically redistribute on dsDNA by 1D diffusion like other SMC complexes.[26,27,30,31] For this purpose, after loading the protein on double-tethered curtains under the same conditions as for the lifetime measurements, we exchanged the running buffer to buffer containing increased salt concentrations (Figure 2A). We found that Smc5/6 was fairly static at low monovalent salt concentrations, but displayed diffusive behavior with increasing diffusion coefficients at higher salt concentrations (Figures 2B and 2C), suggesting that the movement of complexes may be hindered by electrostatic interactions with DNA, as previously observed for other SMCs.[27,32]

We further observed that Smc5/6 showed end-dependent dissociation when it was first bound to DNA in a single-tethered curtain (Figure 2D) and then subjected to a chase with higher salt concentration (Figure 2E). While the binding position of complexes before salt arrival again reflected the center-preference highlighted earlier (Figure 2F), Smc5/6 complexes preferentially dissociated at the free DNA end (Figure 2G), suggesting a topological entrapment of the DNA by the complex. Nevertheless, a smaller fraction of Smc5/6 dissociated from the DNA at internal sites, indicating that not all have converted to a topologically bound configuration. In agreement with a topological binding model, Smc5/6 complexes that move on double-tethered DNA at 250 mM NaCl displayed much longer lifetimes than complexes bound to topologically unconstrained single-tethered DNA under the same salt conditions (Figure S2A), suggesting that the absence of a free DNA end impedes dissociation. Furthermore, in the topologically constrained double-tethered case, buffer flow pushed complexes towards the downstream barrier, but complexes did not dissociate (Figure S2B). These experiments

recapitulate previous findings with cohesin.[27] In summary, Smc5/6 complexes dynamically move along DNA by 1D diffusion and overwhelmingly display minutes-long lifetimes on double-tethered DNA, but readily dissociate once they encounter a free end, indicating that Smc5/6 interacts topologically with DNA[22] like cohesin.[27,33]

Simultaneous binding of ssDNA and dsDNA

Intrigued by our initial observation that Smc5/6 preferentially localizes to the A/T-rich center of dsDNA, which is prone to local melting,[28,29] we next tested whether Smc5/6 could also directly be recruited to ssDNA. To this end, we produced long tracts of ssDNA by rolling-circle replication from a circular M13 DNA substrate by Φ29-polymerase[34] (Figure 3A) and studied the binding behavior of Smc5/6 on this substrate. Confirming our suspicion, Qdot-labeled Smc5/6 complexes readily bound to ssDNA (Figure 3B), but showed no sequence-specific binding preference (Figure 3C).

Having observed that Smc5/6 binds to both dsDNA and ssDNA, and with Smc5/6's role in telomere maintenance and DNA repair in mind, we next asked if Smc5/6 can bind both substrates simultaneously. To test this, we first loaded unlabeled Smc5/6 onto a double-tethered curtain of dsDNA and then flushed in fluorescently tagged ssDNA oligos (cyan, Figure 3D). Intriguingly, we observed the rapid appearance of puncta on the dsDNA, corresponding to the capture of ssDNA oligos. To confirm that the capture of oligos was specific to locations where Smc5/6 was bound, we subsequently flushed in a Qdot label specific to Smc5/6. We observed Qdot localization to sites that had bound fluorescent oligos (Figure 3E). This capture of oligos strictly required the prior loading of Smc5/6 (Figures 3G and S3A). Our observations indicate that Smc5/6 can simultaneously bind to dsDNA and ssDNA.

Capture of dsDNA in *trans* and in *cis*

When we repeated the ssDNA capture experiment with fluorescently tagged dsDNA as a capture target (cyan, Figure 3D), we were surprised to find that dsDNA oligos were recruited to dsDNA-bound Smc5/6 complexes as well (Figure 3F). As for ssDNA, this recruitment was also dependent on the presence of Smc5/6 (Figures 3G and S3B). The location of captured dsDNA puncta was not exclusively at the center of the tethered substrate (Figure 3H), ruling out the possibility that the observed events constitute dsDNA that was recruited to Smc5/6 bound to melted ssDNA at the λ-DNA center. We hence conclude that Smc5/6 cannot only interact with both dsDNA and ssDNA simultaneously but can also interact with multiple dsDNA molecules in *trans*.

Proteins that can interact with multiple dsDNA molecules are expected to form DNA loops and are therefore likely to compact DNA. Confirming this prediction, Smc5/6 readily bound to and compacted single-end-tethered flow-stretched DNA (Figures S3G–S3I). Interestingly, the compaction occurred in a flow-rate-dependent manner with no compaction at flow rates $\geq$20 μL/min (Figure S3I). These results suggest that the compaction is driven by the Smc5/6-mediated capture of thermally driven DNA loop collisions, rather than active compaction.

We next asked whether the recruitment of ssDNA and dsDNA in *trans* is limited to single molecules or if Smc5/6 can recruit multiple DNA molecules. We therefore repeated the capture experiments and recorded photobleaching trajectories of single puncta after flushing out unbound DNA (Figure 3H). To our surprise, ~79% (303/387) of all trajectories of captured ssDNA photobleached in more than a single step, showing that the fluorescent puncta in fact contain multiple captured ssDNA molecules (Figure 3I). To quantify the stoichiometry, we estimated the number of bound ssDNA molecules from the number of photobleaching steps in each trajectory (Figures S3C and S3D). Confoundingly, the distribution of captured oligos was not peaked at a unique value but rather wide (Figure 3J), with a mean of ~4.0 oligos per punctum. In addition, a non-negligible fraction of puncta contained six or more oligos. The wide distribution disagrees with a scenario where the observed capture events are caused by single Smc5/6 molecules featuring a well-defined number of DNA binding sites. Instead, the data are compatible with either the unlikely scenario that Smc5/6 features more than six additional DNA binding sites, or the likelier scenario that Smc5/6 itself exists in oligomeric form.

When we repeated the same experiment for dsDNA instead of ssDNA, we found multi-step photobleaching in ~64% of trajectories (143/222) (Figures 3K, S3E, S3F). As for ssDNA, we also observed a wide distribution with a maximum of 8 and a mean of ~3.2 dsDNA molecules per punctum (Figure 3L). Substitution of ATP with ATPγS resulted in similar observations, suggesting that ATP hydrolysis is not required for the capture of dsDNA. As above, these data indicate that either Smc5/6 features nine or more DNA binding sites or that it exists in oligomeric form. These results, taken together, show that Smc5/6 can link multiple dsDNA and ssDNA molecules.

Oligomerization in solution and on DNA

The ability of Smc5/6 to bind to multiple molecules of ssDNA and dsDNA raises the question of stoichiometry, i.e., is Smc5/6 monomeric? To address this question, we tagged Smc5/6 with a mixture of cyan- and magenta-colored Qdots targeting the same epitope to exclude double labeling and tested its binding to surface-bound dsDNA (Figure 4A). While many binding events were only detected in one camera channel, to our surprise we detected a synchronous appearance of cyan and magenta signal in ~39% (56/141) of binding events from solution (Figure 4B, left), which indicates that these complexes exist in solution at least in a dimeric form. In addition, we also detected a sizeable fraction of complexes (~10%) that oligomerized on DNA (Figure 4B, right), i.e., they bound to previously bound Smc5/6. Clearly identified oligomers, i.e., Smc5/6 that are labeled with two colors, showed the same properties as Smc5/6 that only acquired one color, including diffusion at high salt concentration (Figure 4C). We conclude that most—if not all—fluorescent puncta observed in our experiments represent oligomeric Smc5/6.

To determine the stoichiometry of Smc5/6 oligomers in solution, we counted the fraction of magenta-only, cyan-only, or dual-color puncta, as they first appeared on DNA (Figure 4D, see Figure 4B for kymograms). We next used statistical inference (see STAR Methods) to determine the expected fraction of these values, under the assumption that all oligomers have the same stoichiometry (Figures S4A–S4C). A higher number

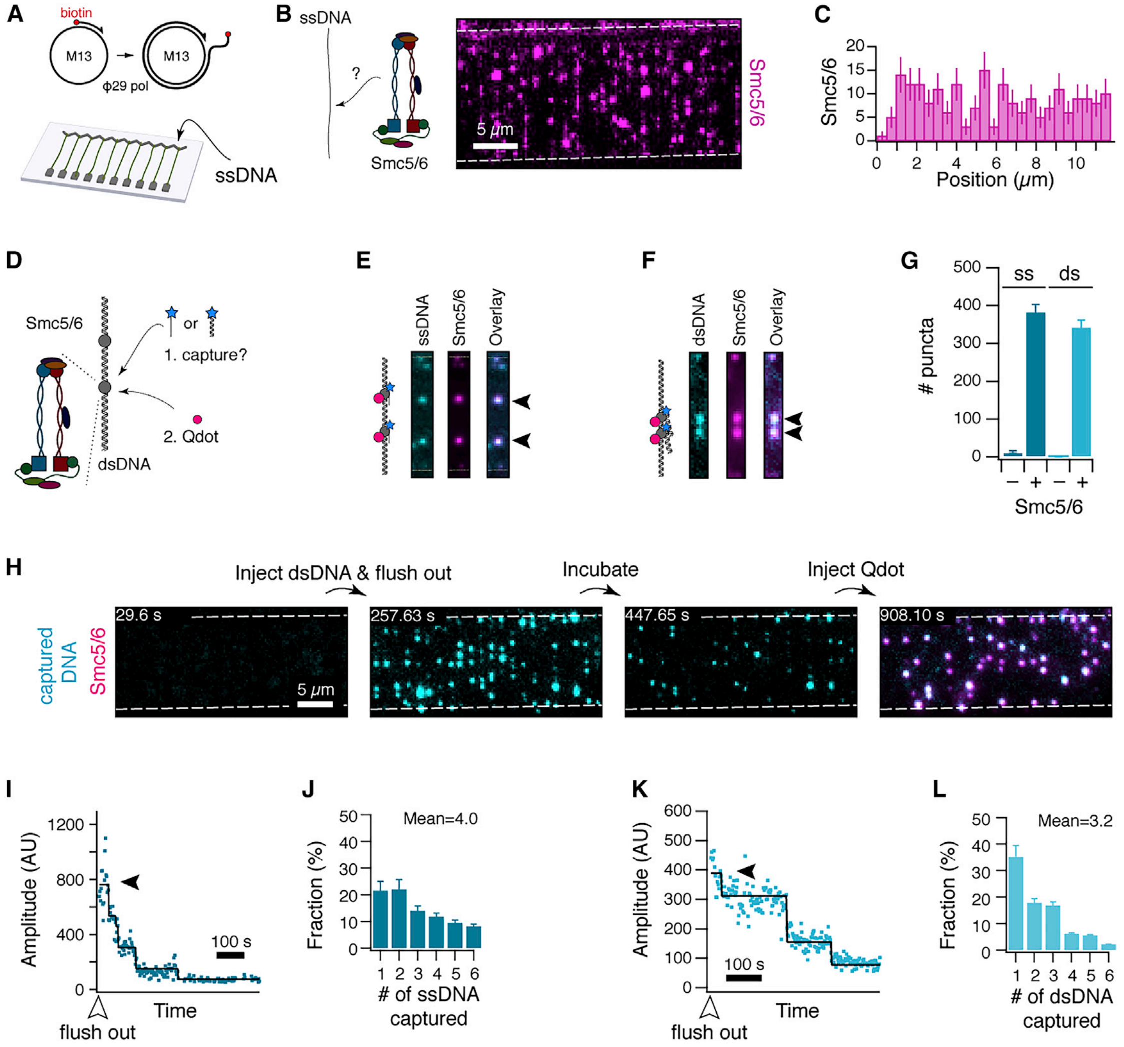

Figure 3. Smc5/6 interacts with ssDNA and dsDNA simultaneously

(A) Schematic of ssDNA production by rolling circle replication off a circular M13 substrate and its assembly into double-tethered ssDNA curtain.

(B) Wide-field image of Smc5/6 (magenta) bound to ssDNA (unlabeled).

(C) Histogram of binding positions of Smc5/6 on ssDNA (n = 209).

(D) DNA curtains experiment to test for capture of DNA oligonucleotides. Dark Smc5/6 was loaded onto a double-tethered DNA curtain followed by injection of fluorescently end-labeled oligonucleotides. As a second step, a Qdot label (magenta) targeting Smc5/6 is injected.

(E) Example image of a ssDNA capture experiment outlined in (D).

(F) Experiment as in (E), but with dsDNA instead of ssDNA as a capture target.

(G) Quantification of the number of fluorescent puncta corresponding to captured ssDNA or dsDNA in the absence or presence of Smc5/6.

(H) Example wide-field view of a time-course of a dsDNA capture experiment.

(I) Example of fluorescence trajectory of a punctum in an ssDNA capture experiment after flush-out of unbound ssDNA, showing photobleaching of ssDNA labels in multiple discrete steps. The amplitude after flushing out the ssDNA (black arrow) provides information on the number of captured ssDNA molecules in a punctum.

(J) Histogram of the number of captured ssDNA molecules per punctum based on single-step photobleaching trajectories (n = 387) see (I).

(K) Fluorescence photobleaching trajectory as in (I), but for the capture of dsDNA.

(L) Histogram of captured dsDNA molecules based on single-step photobleaching trajectories (n = 222), see (K). Data were collected from at least 40 DNA molecules. Error bars represent 68% confidence intervals. See also Figure S3.

of Smc5/6 per punctum is expected to produce a higher number of dual-color detections and vice versa. The predicted values as a function of average cluster size are shown as continuous lines in Figure 4D. By comparing the predictions with the experimentally determined ratio of detected colors (shaded regions in Figure 4D), we find that the average cluster size is about two Smc5/6 complexes per punctum. A more rigorous analysis using a maximum likelihood estimator (see STAR Methods) arrives at the same conclusion (black line in Figure 4E).

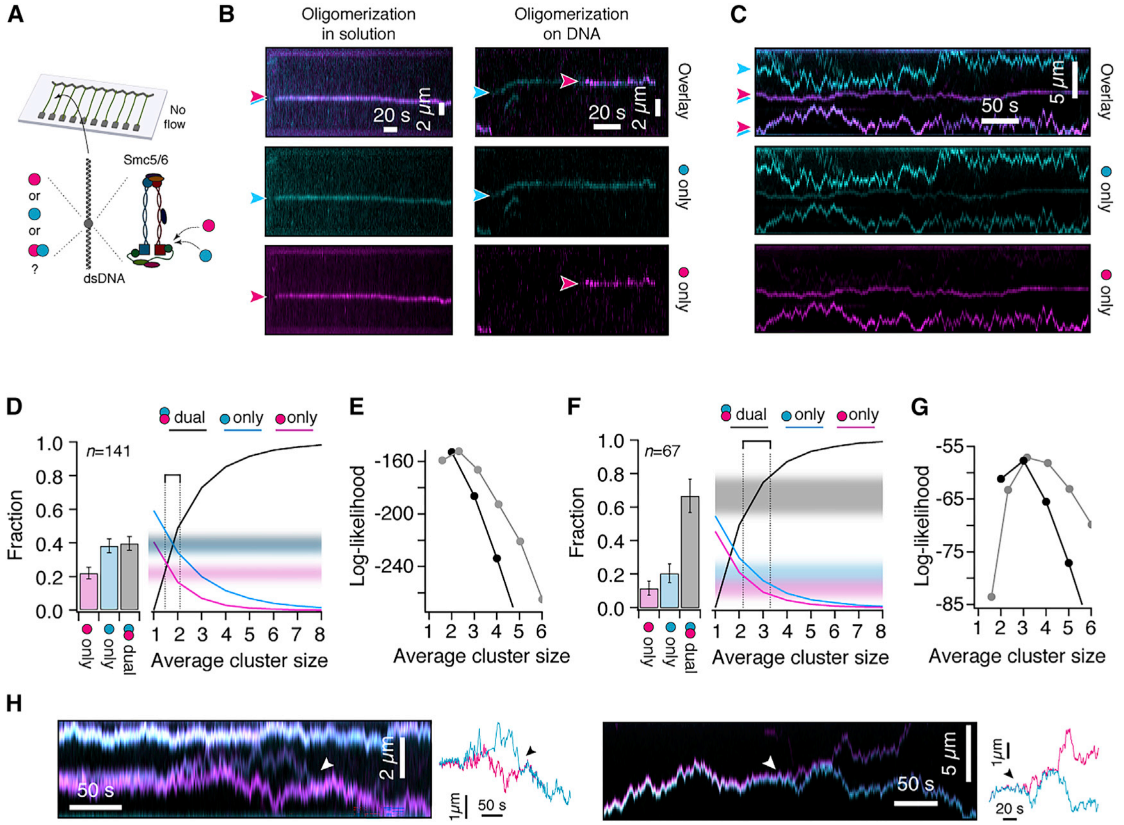

Figure 4. Smc5/6 forms oligomers in solution and on DNA

(A) Assay to study protein oligomerization. Smc5/6 complexes were stochastically labeled with Qdots of two different colors to the same epitope and their binding was visualized using double-tethered DNA curtains.

(B) Example kymograms where oligomeric Smc5/6, i.e., labeled with two different colors, binds DNA from solution (left) or where binding occurs at sites of previously bound Smc5/6 (right).

(C) Example kymogram of Smc5/6 behavior at 250 mM NaCl, indicating that many observed puncta contain at least two labels, i.e., are oligomers.

(D) Quantification of the fraction of magenta-only, cyan-only, and dual-color binding events (n = 141) in the experiment of (B). Continuous lines in the right graph represent the expected ratios as a function of cluster size. The intersection of the model lines with the observed values (shaded intervals) indicates an average cluster size of ~2 molecules/punctum.

(E) Log-likelihood function of the data in (D) as a function of average cluster size, comparing a model where every punctum contains the same number of monomers (black) to a model where the oligomeric state in a punctum follows a model truncated Poissonian distribution (gray). See STAR Methods for details.

(F) Quantification of high-salt oligomerization on DNA (n = 67) in the experiment of (C) and model comparison as in (D). Data were collected from at least 40 DNA molecules. Error bars represent 68% confidence intervals.

(G) Log-likelihood function (E) of high-salt oligomerization indicates an average cluster size at high salt of ~3–4 molecules/punctum.

(H) Example kymograms of dynamically dissociating (left, arrowheads) or re-associating (right, arrowheads) DNA-bound oligomers at 250 mM NaCl. See also Figure S4.

When we repeated the same analysis for Smc5/6 that diffused freely on DNA at higher salt concentrations, we found that the fraction of puncta with dual color increased significantly from ~39% to ~67% (45/67) (Figure 4F), consistent with the notion that Smc5/6 oligomers also coagulate on DNA (cf. Figure 4B). Using the same statistical method as described before, we find that the detected ratios of magenta-only, cyan-only, and dual-color puncta are compatible with an average cluster size of about 2–3 Smc5/6 per punctum (Figures 4F and 4G).

To validate the robustness of the cluster size prediction, we applied an alternative model assuming that the number of Smc5/6 per punctum follows a wider distribution, e.g., a truncated Poissonian (Figures S4D–S4F). Comparable with the first model, we find that the inferred average cluster size of Smc5/6 is ~2–3 in solution (gray line in Figure 4E), and ~3–4 after equilibration on DNA (Figure 4G). We note that all estimates of stoichiometry are based on the assumption that there are no unlabeled complexes. The reported stoichiometries should therefore be regarded as a lower limit.

In addition to the described dynamics of Smc5/6 cluster association on DNA, where Smc5/6 clusters can combine into larger ones, we also observed the reverse process, i.e., the dissociation of oligomers during diffusive scanning on DNA (10 events observed on 41 kymograms) (Figure 4H). Our results provide evidence that Smc5/6 forms oligomers in solution that

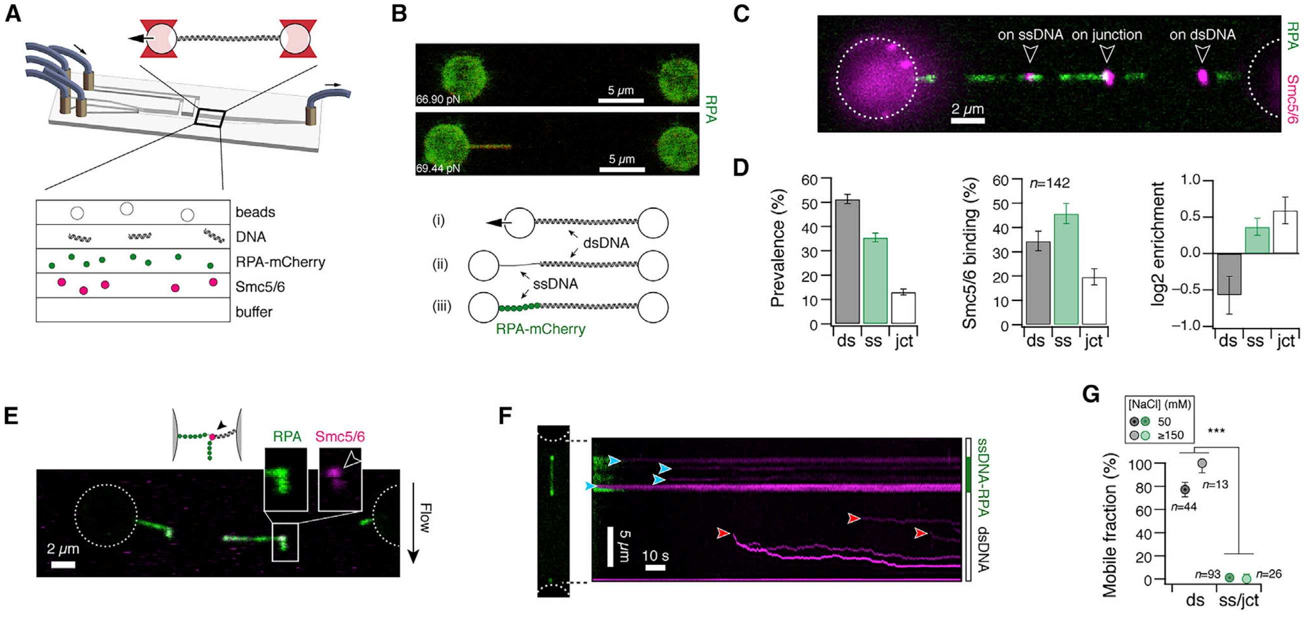

Figure 5. Smc5/6 associates with ssDNA-dsDNA fork junctions

(A) Microfluidics flow chamber and optical tweezers setup.

(B) Stretching of dsDNA by optical tweezers beyond the melting transition exposes ssDNA around nick sites which is rapidly covered by RPA-mCherry (green).

(C) Schematic and wide-field image of Smc5/6 (magenta) bound to stretched hybrids of dsDNA and ssDNA-RPA.

(D) Quantification of the prevalence of dsDNA, ssDNA-RPA tracts and junctions (left) from wide-field imaging (see Figure S5A). Classification of binding events (n = 142, middle) into dsDNA-bound, ssDNA-bound, or junction bound, and the relative enrichment (right) at these sites. Data were collected from 35 tethers. Error bars represent 68% confidence intervals.

(E) Flow application perpendicular to the DNA stretches out unpeeled RPA-coated ssDNA (green). Smc5/6 (magenta) is predominantly bound to the ssDNA-dsDNA fork junction.

(F) Wide-field image and kymogram of Smc5/6 binding to ssDNA-dsDNA hybrids. Complexes in ssDNA regions are static (blue arrows), while those in dsDNA regions are mobile (red arrows).

(G) Quantification of the fraction of mobile complexes, depending on their binding substrate and the salt concentration. Data were collected from at least 10 tethers. Significance was determined by Fisher's exact test. See also Figure S5.

diffusively translocate along dsDNA. Diffusively scanning oligomeric complexes can dynamically merge or split while bound to DNA.

Smc5/6 is enriched at ssDNA-dsDNA junctions

As a protein implicated in DNA repair, we surmised that Smc5/6's ability to interact with both ssDNA and dsDNA may help its recruitment to lesions on DNA, which often are characterized by the presence of both ssDNA and dsDNA. We tested this using a combined optical tweezers and confocal microscopy assay, which allowed us to force-manipulate single molecules of λ-DNA during concurrent imaging in a microfluidics chamber with multiple laminar flow channels (Figure 5A). We generated hybrids of ssDNA and dsDNA by force melting, which exposes ssDNA around random nick sites along the DNA (Figure 5B (i)–(ii)). Mimicking *in vivo* conditions, where unprotected ssDNA is rapidly sequestered by single-stranded binding proteins,[35] we exposed the molecule to RPA-mCherry, which also allowed us to visualize the ssDNA sections (Figure 5B (iii), green).

We then tested whether Smc5/6 gets preferentially recruited to the dsDNA sections (dark), ssDNA sections (green), or the junctions in between (Figure 5C). Corroborating our observations on DNA curtains that Smc5/6 interacts with both dsDNA and ssDNA, we found that ∼35% (49/142) of complexes were bound to dsDNA and ∼45% (65/142) were bound to ssDNA. In addition, ∼20% (28/142) were bound to ssDNA-dsDNA junctions (Figure 5D, middle). To assess whether Smc5/6 preferentially targets any of these substrates, we then measured the available length of dsDNA and ssDNA stretches by imaging (Figures S5A and 5D, left) and determined the resulting enrichment on dsDNA, on ssDNA, and on junctions, by counting the number of binding events relative to the available length of DNA substrate (see STAR Methods). We found that Smc5/6 was relatively depleted on dsDNA but enriched on ssDNA as well as on ssDNA-dsDNA junctions (Figure 5D, right).

Smc5/6's association with both ssDNA and dsDNA indicates that binding events on junctions represent simultaneous binding to ssDNA and dsDNA regions. However, it is also conceivable that on-junction events are solely bound to the tether ssDNA or the unpeeled ssDNA. To distinguish these scenarios, we recorded images of Smc5/6 binding to hybrid substrates with buffer flow perpendicular to the DNA. As expected from our previous experiments, we find examples of all scenarios: binding to the junction (Figure 5E), as well as binding to the ssDNA tract (Figures S5B and S5C). However, we note that the scenario of direct binding to the junction as well as direct binding to ssDNA

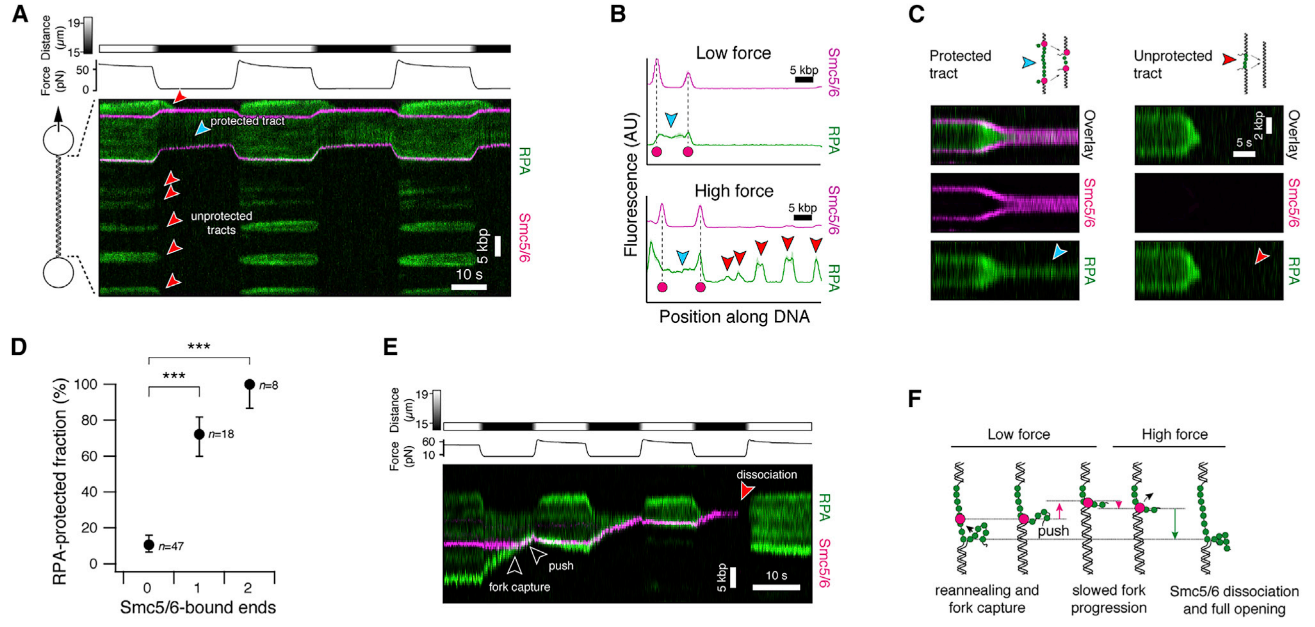

Figure 6. Smc5/6 stabilizes ssDNA-dsDNA fork junctions

(A) Kymogram of tracts of RPA and Smc5/6 during force manipulation. Red arrowheads identify tracts of RPA that are not flanked by Smc5/6 (unprotected) and show complete RPA eviction at low force. The blue arrowhead identifies an RPA tract that is flanked on both sides by Smc5/6. This tract remains RPA bound even at low force.

(B) Fluorescence profiles of the tether in (A) at low force and high force. Red arrowheads denote unprotected tracts that disappear at low force, blue arrowhead denotes a protected tract.

(C) Other examples of protected and unprotected tracts, also showing that Smc5/6-stabilized fork junctions are not fixed in place but can move.

(D) Quantification of the fraction of RPA tracts (n = 71) that are protected (i.e., retain RPA) at low force, depending on whether none, one or both ends are occupied by Smc5/6. Data were collected from at least 10 DNA molecules. Error bars are 68% confidence intervals. Significance was determined by Fisher's exact test.

(E) ssDNA-bound Smc5/6 can be captured by fork junctions, which affects their dynamic behavior. While the RPA tract size is limited during junction association, its size suddenly increases after Smc5/6 has dissociated. Also see Figures S6F and S6G.

(F) Cartoon interpretation of (E). See also Figure S6.

is unlikely to be the preferred recruitment mode in a physiological setting, where dsDNA is much more abundant than ssDNA. We therefore conducted additional experiments to reveal how Smc5/6 associates with fork junctions.

Differential dynamics on dsDNA and ssDNA cause enrichment on ssDNA-dsDNA junctions

The previous experiments have revealed a significant enrichment of Smc5/6 at ssDNA-dsDNA fork junctions, a likely precursor for activation in DNA repair.[1] To understand whether the recruitment of Smc5/6 to junctions is driven by direct binding from solution or 1D scanning along the DNA, we next recorded kymograms of Smc5/6 translocation dynamics after their association to ssDNA-dsDNA hybrids (Figure 5F). While Smc5/6 generally showed diffusive behavior on dsDNA stretches (red arrows), as previously shown on DNA curtains, proteins bound to the ssDNA portion were overall static and without detectable movement (blue arrows). We visually classified trajectories into mobile or static, depending on their binding substrate. At 50 mM NaCl, ~77% (34/44) of dsDNA-bound complexes were mobile, while only ~1% (1/93) were mobile on ssDNA (Figure 5G). To avoid misclassification of slowly diffusing complexes into the static class, we repeated the experiment at 150 mM NaCl to increase the diffusion speed (see Figures 2B and 2C) and found the same clear difference in translocation behavior between ssDNA and dsDNA (Figure 5G).

In vivo, RPA is abundant[35] and ssDNA is therefore unlikely to appear in its naked form. Nevertheless, we still wondered if Smc5/6's static binding to ssDNA may be a consequence of hindered diffusion due to blockage by the presence of filamentous RPA, or if Smc5/6 is also static on naked ssDNA. To address this question, we recorded the translocation dynamics of Smc5/6 on ssDNA-dsDNA hybrids without RPA present. We found that Smc5/6 diffuses on DNA at low tension, when tethers contain only little ssDNA, but converts into a static form at high tension, when tethers contain more ssDNA (Figure S5D).

The drastically different dynamics of Smc5/6's motion on dsDNA compared with ssDNA suggest that Smc5/6 DNA repair complexes scan dsDNA by 1D diffusion and are stopped by static association to fork junctions, leading to recruitment to DNA lesions.

Smc5/6 stabilizes ssDNA-dsDNA fork junctions and RPA tracts

After characterizing the mechanism of Smc5/6 enrichment to junctions of ssDNA and dsDNA, we next investigated the downstream consequences of this association. ssDNA-dsDNA junctions can occur in a physiological setting by a variety of pathways

CellPress
OPEN ACCESS

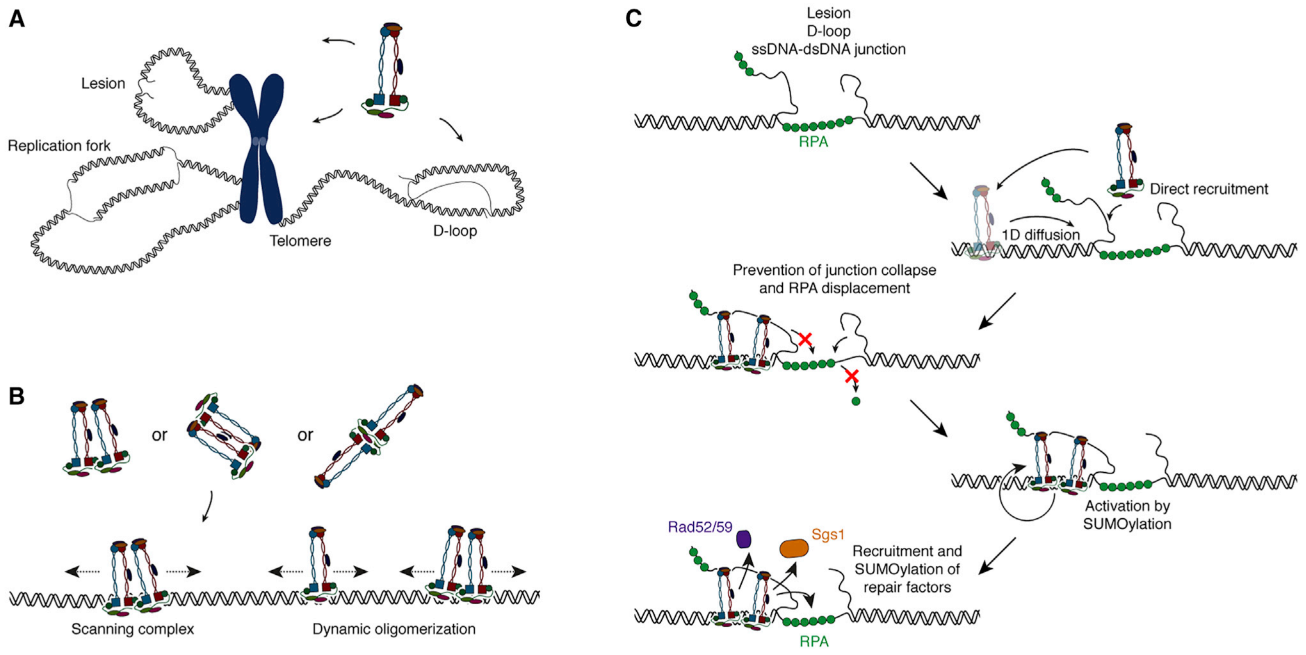

Figure 7. The recruitment of Smc5/6 to ssDNA-dsDNA junctions is important for its biological function

(A) Smc5/6 is implicated in chromosomal maintenance on various substrates that feature the presence of both ssDNA and dsDNA.

(B) Model for the recruitment of oligomeric Smc5/6 to target sites either through direct binding of Smc5/6 oligomers from buffer or through diffusive scanning. Our experiments provide evidence for oligomerization in buffer as well as for a dynamic redistribution of the oligomerization state on DNA.

(C) Model for Smc5/6's function in the stabilization of ssDNA-dsDNA forks and its subsequent SUMO-mediated recruitment and activation of ancillary repair factors.

and specifically occur as intermediates of HR. In this context, RPA-bound ssDNA is a necessary intermediate for the formation of HR-active presynaptic filaments.[9,10] We therefore returned to the force-melting assay to investigate how Smc5/6 influences the stability of RPA tracts. After force-melting, we exposed tethers to RPA and Smc5/6 and altered the tether tension by stepping the inter-trap distance between ~15 and ~20 μm (Figure 6A). This resulted in a tether tension of ~65 pN at high distance and ~5 pN at low distance. As previously, high forces across the tether exposed ssDNA tracts that were rapidly covered by RPA (Figure 6A, green). When the force was lowered, almost all RPA tracts disappeared, indicating that reannealing of the displaced ssDNA results in the eviction of RPA (red arrows). Raising the force caused the RPA tracts to reappear, corroborating our interpretation that RPA tracts form by local unpeeling of ssDNA around nicks. These observations indicate that RPA tracts are inherently unstable in a physiological low-tension setting and require additional factors that either resect the competing strand or stabilize the junction in a different way to prevent RPA eviction.

Intriguingly, we observed that RPA tracts that were flanked by Smc5/6 complexes at their junctions to dsDNA regions resisted RPA eviction (blue arrow) and remained RPA covered during repeated force cycles. A quantitative fluorescence profile of the kymogram of Figure 6A at low and high forces confirms that RPA tracts without flanking Smc5/6 are completely dissolved (red arrows in Figure 6B), but the tract bound on both sides (blue arrow) remains protected. Figure 6C shows further examples of doubly Smc5/6-flanked RPA tracts (left), which remain open (blue arrow), and of unbound RPA tracts (right), which are dissolved (red arrow).

To quantify whether the protection of RPA tracts correlates with Smc5/6, we counted the fraction of RPA tracts that resisted eviction at low force (protected), depending on whether none, one, or both termini were occupied by Smc5/6. While tracts that were not flanked by visibly labeled Smc5/6 were protected in ~11% (5/47) of cases, strikingly the protected fractions were significantly higher in the case of one occupied terminus (~72%, 13/18) or two occupied termini (100%, 8/8) (Figure 6D). In conclusion, Smc5/6 stabilizes ssDNA-dsDNA fork junctions and protects against the removal of RPA, which is a required intermediate step in many DNA repair processes. Our data suggest that this protection function is caused by Smc5/6's ability to interact with both ssDNA and dsDNA portions of the fork, which prevents RPA eviction due to reannealing.

A rigid stabilization of junctions by Smc5/6 may be detrimental to downstream repair processes, where processing machinery must remove Smc5/6 at some point. We therefore asked if junctions stabilized by Smc5/6 are static or allow for sliding of the junction fork. We tracked the RPA tract termini of unprotected (Figure S6B) and protected tracts (Figure S6C). As RPA-covered ssDNA and dsDNA have widely different persistence lengths, the directly accessible coordinate of molecular extension is not a good reporter on the true tract contour length. Instead, we used polymer models to determine the contour length of the tracts (STAR Methods; Figure S6A). As previously, most

unprotected RPA tracts were rapidly dissolved at low force through strand reannealing (arrowheads in Figure S6D). Strikingly, however, we found that Smc5/6-stabilized RPA tracts were not completely static but allowed for junction sliding and slow strand reannealing (arrowheads in Figure S6E).

Similarly, we observed that Smc5/6 also associates with fork junctions when it is initially bound to ssDNA regions (Figures 6E and 6F, also see Figures S6F and S6G). Upon encountering and capturing the fork junction, Smc5/6 is pushed along the DNA. During high-force periods, the Smc5/6-bound RPA tract is kept from returning to its initial size, implying that Smc5/6 stabilizes the fork. In addition, the complex slows the reannealing of the displaced strand and removal of the RPA tract during low-force periods. After the stochastic dissociation of Smc5/6, the RPA tract increases in size. We conclude that Smc5/6 can localize to junctions via diffusion on dsDNA where it stabilizes RPA tracts by slowing its ssDNA-reannealing-mediated eviction.

Taken together, our results imply that Smc5/6 is recruited to and stabilizes ssDNA-dsDNA fork junctions which we artificially produced using force melting (Figure S6H), but which occur *in vivo* in a variety of processes in a physiological setting, such as during HR, on stalled replication forks, or at telomeres. Through direct association to and stabilization of the fork junction, Smc5/6 prevents the eviction of RPA. We speculate that, in addition to preventing the eviction of RPA, Smc5/6's SUMOylation activity may then lead to the recruitment and activation of downstream repair factors.

DISCUSSION

The Smc5/6 complex, like its SMC siblings cohesin and condensin, plays a major role in the organization and maintenance of chromosomes. While there is a general consensus on the salient roles of condensin and cohesin, the former being implicated in the condensation of chromosomes, the latter in the establishment of topologically associating domains and sister chromatid cohesion,[3] Smc5/6's principal role remains elusive to date. Nevertheless, the complex has been recognized as a DNA repair protein involved in the repair of DSBs, the recovery from stalled replication forks as well as the maintenance of telomeres, in part through its coordinative roles in HR-associated processes.[5,13,18] Homologous recombination can lead to toxic intermediates that need to be processed before chromosome segregation. Smc5/6 regulates, through an unclear mechanism, the proper removal of these intermediates both in the context of meiotic recombination[20] and of challenged replication forks at DNA damage sites or of replication stress.[1,12] Smc5/6's roles at replication forks extend beyond resolution of intermediates, with the complex implicated in the incipient phase of recombination intermediates generation as well.[13,14]

Intriguingly, the structural features of intermediates associated with these processes share the same hallmarks: close proximity of dsDNA to exposed ssDNA or ssDNA-RPA (Figure 7A). This has led to proposals that Smc5/6's role in these repair pathways may be defined by its ability to recognize lesion-associated DNA structures[20] and activate downstream repair responses, for example, through the recruitment and activation of intermediate-removing helicases, such as Sgs1 or Mph1.[1]

Our experiments demonstrate a potential mechanism of how Smc5/6 may be recruited to stress intermediates and prepare them for an efficient downstream repair response, illustrating how observable molecular properties can predict a likely mechanism of Smc5/6's involvement in DNA repair.

A complex scanning for lesions

Diffusive motion on dsDNA and recruitment to lesions

While the importance of Smc5/6 in the repair of DNA lesions has been well described in genetic screens, the mechanism of how it is recruited to these sites is less clear. Our experiments revealed that Smc5/6 translocates by 1D diffusion along dsDNA but statically interacts with exposed ssDNA (Figure 5F). This mechanism provides a simple explanation for how Smc5/6 accumulates at lesions that are characterized by a close proximity of dsDNA and ssDNA. *In vivo*, exposed ssDNA is rapidly covered and protected by single-stranded binding proteins, such as the bacterial SSB or the eukaryotic RPA. Corroborating the proposed mechanism, coverage of ssDNA by RPA did not prevent binding of Smc5/6 (Figure 5C). This finding confirms predictions made on the complex's interaction with RPA-covered ssDNA in previous studies.[21,36,37]

A mechanism of diffusive search on dsDNA may not be unique to Smc5/6 in the world of SMC complexes. In fact, diffusive motion on dsDNA has been observed for other eukaryotic SMC proteins, such as cohesin[27,32] or condensin.[30] The prokaryotic bsSMC shows both diffusive and static motion on dsDNA.[38] A similar bivalent motion mechanism has also been shown for the isolated cohesin subunit Scc3/SA2, which diffusively translocates on dsDNA and statically interacts with ssDNA regions.[39] Furthermore, a comparable diffusion search mechanism is employed by other proteins involved in DNA repair, such as the mismatch repair protein MutSα.[40] We propose that, for Smc5/6, different dynamics of interaction with dsDNA or ssDNA allows the complex to scan along dsDNA until it encounters a lesion, defined by a ssDNA-dsDNA interface. Stable binding of the complex marks this interface for further processing.

Corroborating this model, *in vivo* studies have suggested that dsDNA binding is required for association with chromatin, while ssDNA association is not.[41] Inactivation of the ssDNA interaction at the hinge leaves the cell sensitive to replication stress and DNA damage, but Smc5/6 chromatin loading in unperturbed cells remains unchanged,[42] suggesting that ssDNA binding may be necessary only in the complex's coordinating role in recombination but not for chromatin association.[41] Based on these observations we propose that, *in vivo*, Smc5/6 recruitment to the ssDNA-dsDNA junction occurs through, first, Nse3-dsDNA-interaction-driven loading on chromatin,[41,43] followed by free diffusion on dsDNA until encounter of the ssDNA-dsDNA interface.

Nucleotide dependence

The crucial role of nucleotide turnover in the complex's functionality is emphasized by its ATPase activity, which dictates both its efficient association on chromatin[41] and the activation of the SUMO ligase which triggers recruitment of repair factors containing SIM domains.[15,16]

In our single-molecule setup, the Smc5/6 holocomplex shows binding to dsDNA largely independently of nucleotide presence,

in line with previous bulk investigations of the Smc5/6 dimer[21] or the holocomplex.[22] In addition, we show that nucleotide identity seems to play a role in regulating the binding dynamics (Figure 1D). In the absence of nucleotide, or in the presence of ATPγS, Smc5/6 exhibited long lifetimes, while ADP or ATP moderately but measurably increased the off-rate of the complex. The enhanced binding observed in absence of ATP corroborates the outcome of a pull-down study investigating stable salt association with circular DNA of the complex,[44] where lower salt buffer wash led to a slightly enhanced Smc5/6 binding in the nucleotide-free state. Based on these observations, we speculate that ATP hydrolysis makes the complex dissociate and be recycled or that it allows it to come off when it does not lock into the right substrate.

Confoundingly, the ATPγS-bound complex exhibited an increased number of DNA binding events, similar to observations of enhanced retention of Smc5 on ssDNA under the same nucleotide state.[45] The ATPγS-bound state is characterized, according to crosslinking studies, by a lack of head engagement.[44] We speculate that, in this head-disengaged state, the complex may be prone to enhanced DNA binding—in particular to partially melted structures.

Nucleotide state has been shown to determine also the topological nature of the DNA interaction. Our single-molecule investigation, using linear DNA and in the presence of ATP, permitted the direct visualization of topologically associated Smc5/6: dissociating from a free DNA end (Figure 2E) or being pushed without dissociation in a double-tethered DNA setup (Figure 2SB). Bulk pull-down studies with circular DNA substrates have also indicated that the complex can bind topologically in ATP-dependent manner, the hexamer complex stably associating with both nicked and negatively supercoiled plasmid at high salt,[46] while the octamer was further shown to topologically entrap positively supercoiled substrates as well as kinetoplast DNA.[22]

Oligomerization

In this work, we demonstrated that Smc5/6 oligomerizes both in solution and on DNA, with a minimum number of two Smc5/6 per oligomer. Crucially, we observed that DNA-associated scanning Smc5/6 oligomers can merge and dissociate, indicating that the oligomerization likely occurs in a dynamic equilibrium (Figure 4). Whether proteins involved in DNA repair act as monomers or self-associate into oligomeric forms is an ongoing topic of investigation. Similar to Smc5/6, DNA repair processing complexes such as the SMC-like Mre11-Rad50 (MR) complex[47] or the recombination mediator BRCA2[48] have recently been shown to have a biologically active oligomeric state. Interestingly, oligomerization has also been reported for related proteins of the SMC family, such as condensin,[49] cohesin,[50] or MukBEF.[51] We therefore speculate that oligomerization may be a functional feature that is conserved among many proteins involved in chromosomal organization or DNA repair.

The observation of oligomerization warrants a second take on the experimental observation of end-dependent dissociation of complexes (Figures 2D–2G). In previous studies, end-dependent dissociation of diffusive molecules at high salt concentration has suggested that these proteins are topologically bound to DNA, i.e., they encircle the DNA strand.[27,32] For monomeric proteins, this model predicts the presence of a topological opening inside the complex's ring-like structure. However, the clear observation of oligomeric Smc5/6 provides an alternative molecular interpretation of the same end-dependent dissociation observed for Smc5/6 (Figures 2D–2G), where the DNA is still topologically bound, but encircled by multiple Smc5/6 complexes instead of a single complex in a so far unknown topological arrangement.

Compaction of DNA substrates as a general hallmark of repair proteins for recruitment and activation

In line with previous observations carried on a magnetic tweezers setup,[22,23] Smc5/6 in our DNA curtains assay was able to compact dsDNA at low tension (Figures S3G–S3I) and to bind dsDNA in *trans* (Figure 3). Furthermore we show that the complex can link ssDNA and dsDNA molecules (Figure 3). The ability to connect multiple DNA molecules appears to be especially relevant considering the previously discussed difference in movement on dsDNA compared with ssDNA. These observations predict that simultaneous binding to dsDNA molecules is dynamic with sliding movement of DNA relative to Smc5/6. It is conceivable that this mechanism *in vivo* leads to an increase of local DNA concentration that increases the search efficiency for lesions.[22,23]

We envision that, upon encounter with ssDNA around lesion sites, Smc5/6 will lock its movement while retaining a locally compacted environment of DNA as a recruitment platform for additional repair factors. Consequential accumulation of Smc5/6 on ssDNA then likely triggers the activation of Smc5/6's Nse2 SUMO ligase, which is preferentially activated by ssDNA,[52] leading to self-SUMOylation of Smc5/6, SUMOylation of RPA and Rad52/59,[53] and the recruitment and activation of the Sgs1-Top3-Rmi1 (STR) complex, also known as the dissolvasome.[15,16]

Supporting our model of compaction-driven recruitment and activation, strong compaction of DNA and local concentration enhancement of repair factors through the establishment of phase-separated repair foci appears to be a general hallmark of DNA repair,[54,55] meiosis,[56] and the telomeric ALT pathway.[17]

The ability to capture a second DNA molecule has been previously observed for yeast cohesin[26,57] and thus could be a shared feature among SMC molecules.

RPA protection/observation of recruitment to "forks" and consequences

In addition to observing the recruitment process to lesions, our experiments also provide insights into the direct consequences of Smc5/6 binding to hybrid ssDNA-dsDNA structures that resemble the structure of stalled replication forks or HR intermediates, before activation. Interestingly, we observed that, in the absence of Smc5/6, RPA binding at low tension is not able to withstand eviction by reannealing and competition of the displaced strand. However, Smc5/6 association to the ssDNA-dsDNA junction ends of RPA-covered ssDNA tracts stabilizes them and slows down the eviction of RPA, in a mechanism that is likely determined by Smc5/6's binding affinity to both ssDNA and dsDNA. Crucially, while Smc5/6 binding to the fork junction increased the stability of RPA, it did not completely abolish its removal but permitted slow sliding of the junction. Prevention of junction collapse might therefore contribute to maintaining the replication fork in a conformation prone to

replication restart[13] or, in the context of meiotic recombination, constrain D-loop extension and unwanted Holliday junction branch migration.[20]

In summary, our experiments have revealed important aspects of how molecular properties of Smc5/6 complexes contribute to its role as a DNA repair protein in the repair of lesions, the recovery from stalled replication forks and telomere maintenance (Figure 7A). We propose that oligomeric Smc5/6 acts as a marker protein that recognizes lesions/junctions of ssDNA-RPA and dsDNA (Figure 7B) where it stabilizes the junction, creates repair foci by enhancing the local concentration of DNA as a loading platform for additional factors, and, through its Nse2 subunit, activates these additional repair proteins by SUMOylation (Figure 7C).

Limitations of the study

Our work revealed a different mode of interaction of the Smc5/6 complex on ssDNA vs. dsDNA, defining a likely model for its role at sites of stressed replication forks of DNA lesions. Our experiments demonstrate a stable association with ssDNA, which leads to a stabilization of the ssDNA-RPA and dsDNA junctions. Our study nevertheless has limitations. In the context of DNA repair, the Nse2 unit, a SUMO ligase, plays a pivotal role leading to recruitment of additional repair factors. Although our work reports on the SUMOylation capable holocomplex we have not examined how activation of the SUMO ligase impacts the complex's mechanism in DNA repair. It would be interesting in future work to expand on present findings into examining how the activated Smc5/6 enlists downstream repair factors.

STAR★METHODS

Detailed methods are provided in the online version of this paper and include the following:

- KEY RESOURCES TABLE
- RESOURCE AVAILABILITY
  - Lead contact
  - Materials availability
  - Data and code availability statements
- EXPERIMENTAL MODEL AND SUBJECT DETAILS
  - Yeast strains
- METHOD DETAILS
  - Plasmids
  - Expression and purification of Smc5/6 holocomplex
  - mCherry-RPA expression and purification
  - Φ29 polymerase expression and purification
  - DNA curtains setup
  - DNA curtains experiments
  - Diffusion experiments
  - End-dependent dissociation
  - ssDNA binding experiments
  - Second-capture of dsDNA and ssDNA
  - DNA compaction
  - Oligomerization
  - Optical tweezers setup & measurement modes
  - Optical tweezers experiments
- QUANTIFICATION AND STATISTICAL ANALYSIS

SUPPLEMENTAL INFORMATION

Supplemental information can be found online at https://doi.org/10.1016/j.celrep.2022.111778.

ACKNOWLEDGMENTS

The authors thank Dr. Bryan Gibb for sharing the expression plasmid for RPA-mCherry and Φ29 polymerase. We acknowledge technical assistance by Thomas Fröhlich (mass spectrometry) and Simona Nasiscionyte (protein expression). We further thank members of the Stigler lab for comments on the manuscript. This work was supported by the LMU Center for Nanoscience CeNS, a DFG Emmy Noether grant (no. STI673/2-1), and an ERC starting grant (no. 758124).

AUTHOR CONTRIBUTIONS

N.-L.T. conducted the experiments. N.-L.T. and J.S. analyzed the data. N.-L.T., P.G.-E., S.J., and L.A. performed cloning and/or protein expression. N.-L.T. and J.S. wrote the paper with help from all other authors. J.S. supervised the project.

DECLARATION OF INTERESTS

The authors declare no competing interests.

INCLUSION AND DIVERSITY

We support inclusive, diverse, and equitable conduct of research.

Received: March 25, 2022
Revised: September 15, 2022
Accepted: November 11, 2022
Published: December 6, 2022

REFERENCES

1. Aragón, L. (2018). The smc5/6 complex: new and old functions of the enigmatic long-distance relative. Annu. Rev. Genet. *52*, 89–107. https://doi.org/10.1146/annurev-genet-120417-031353.
2. Uhlmann, F. (2016). SMC complexes: from DNA to chromosomes. Nat. Rev. Mol. Cell Biol. *17*, 399–412. https://doi.org/10.1038/nrm.2016.30.
3. Yatskevich, S., Rhodes, J., and Nasmyth, K. (2019). Organization of chromosomal DNA by SMC complexes. Annu. Rev. Genet. *53*, 445–482. https://doi.org/10.1146/annurev-genet-112618-043633.
4. Lehmann, A.R., Walicka, M., Griffiths, D.J., Murray, J.M., Watts, F.Z., McCready, S., and Carr, A.M. (1995). The rad18 gene of Schizosaccharomyces pombe defines a new subgroup of the SMC superfamily involved in DNA repair. Mol. Cell Biol. *15*, 7067–7080. https://doi.org/10.1128/mcb.15.12.7067.
5. De Piccoli, G., Cortes-Ledesma, F., Ira, G., Torres-Rosell, J., Uhle, S., Farmer, S., Hwang, J.Y., Machin, F., Ceschia, A., McAleenan, A., et al. (2006). Smc5-Smc6 mediate DNA double-strand-break repair by promoting sister-chromatid recombination. Nat. Cell Biol. *8*, 1032–1034. https://doi.org/10.1038/ncb1466.
6. Betts Lindroos, H., Ström, L., Itoh, T., Katou, Y., Shirahige, K., and Sjögren, C. (2006). Chromosomal association of the smc5/6 complex reveals that it functions in differently regulated pathways. Mol. Cell *22*, 755–767. https://doi.org/10.1016/j.molcel.2006.05.014.
7. Potts, P.R., Porteus, M.H., and Yu, H. (2006). Human SMC5/6 complex promotes sister chromatid homologous recombination by recruiting the SMC1/3 cohesin complex to double-strand breaks. EMBO J. *25*, 3377–3388. https://doi.org/10.1038/sj.emboj.7601218.
8. Cortez, D. (2019). Replication-coupled DNA repair. Mol. Cell *74*, 866–876. https://doi.org/10.1016/j.molcel.2019.04.027.

CellPress
OPEN ACCESS

9. Kaniecki, K., De Tullio, L., and Greene, E.C. (2018). A change of view: homologous recombination at single-molecule resolution. Nat. Rev. Genet. *19*, 191–207. https://doi.org/10.1038/nrg.2017.92.

10. Kowalczykowski, S.C. (2015). An overview of the molecular mechanismsof recombinational DNA repair. Cold Spring Harb. Perspect. Biol. *7*, a016410. https://doi.org/10.1101/cshperspect.a016410.

11. Nickoloff, J.A., Sharma, N., Taylor, L., Allen, S.J., and Hromas, R. (2021). The safe path at the fork: ensuring replication-associated DNA double-strand breaks are repaired by homologous recombination. Front. Genet. *12*, 748033–748039. https://doi.org/10.3389/fgene.2021.748033.

12. Palecek, J.J. (2018). SMC5/6: multifunctional player in replication. Genes *10*, E7. https://doi.org/10.3390/genes10010007.

13. Irmisch, A., Ampatzidou, E., Mizuno, K., O'Connell, M.J., and Murray, J.M. (2009). Smc5/6 maintains stalled replication forks in a recombination-competent conformation. EMBO J. *28*, 144–155. https://doi.org/10.1038/emboj.2008.273.

14. Zapatka, M., Pociño-Merino, I., Heluani-Gahete, H., Bermúdez-López, M., Tarrés, M., Ibars, E., Solé-Soler, R., Gutiérrez-Escribano, P., Apostolova, S., Casas, C., et al. (2019). Sumoylation of Smc5 promotes error-free bypass at damaged replication forks. Cell Rep. *29*, 3160–3172.e4. https://doi.org/10.1016/j.celrep.2019.10.123.

15. Bermúdez-López, M., Villoria, M.T., Esteras, M., Jarmuz, A., Torres-Rosell, J., Clemente-Blanco, A., and Aragon, L. (2016). Sgs1's roles in DNA end resection, HJ dissolution, and crossover suppression require a two-step SUMO regulation dependent on Smc5/6. Genes Dev. *30*, 1339–1356. https://doi.org/10.1101/gad.278275.116.

16. Wan, B., Arter, M., Matos, J., Torres, N.P., Szakal, B., Wei, L., Bonner, J.N., Choi, K., Xue, X., Sung, P., et al. (2016). Rmi1 complex promotes removal of recombination article Smc5/6 mediated sumoylation of the sgs1-top3-rmi1 complex promotes removal of recombination intermediates. Cell Rep. *16*, 368–378. https://doi.org/10.1016/j.celrep.2016.06.015.

17. Zhang, J.M., and Zou, L. (2020). Alternative lengthening of telomeres: from molecular mechanisms to therapeutic outlooks. Cell Biosci. *10*, 30–39. https://doi.org/10.1186/s13578-020-00391-6.

18. Potts, P.R., and Yu, H. (2007). The SMC5/6 complex maintains telomere length in ALT cancer cells through SUMOylation of telomere-binding proteins. Nat. Struct. Mol. Biol. *14*, 581–590. https://doi.org/10.1038/nsmb1259.

19. Baudat, F., and De Massy, B. (2007). Regulating double-stranded DNA break repair towards crossover or non-crossover during mammalian meiosis. Chromosome Res. *15*, 565–577. https://doi.org/10.1007/s10577-007-1140-3.

20. Xaver, M., Huang, L., Chen, D., and Klein, F. (2013). Smc5/6-Mms21 prevents and eliminates inappropriate recombination intermediates in meiosis. PLoS Genet. *9*, e1004067. https://doi.org/10.1371/journal.pgen.1004067.

21. Roy, M.A., Dhanaraman, T., and D'Amours, D. (2015). The Smc5-Smc6 heterodimer associates with DNA through several independent binding domains. Sci. Rep. *5*, 9797–9812. https://doi.org/10.1038/srep09797.

22. Gutierrez-Escribano, P., Hormeño, S., Madariaga-Marcos, J., Solé-Soler, R., O'Reilly, F.J., Morris, K., Aicart-Ramos, C., Aramayo, R., Montoya, A., Kramer, H., et al. (2020). Purified Smc5/6 complex exhibits DNA substrate recognition and compaction. Mol. Cell *80*, 1039–1054.e6. https://doi.org/10.1016/j.molcel.2020.11.012.

23. Serrano, D., Cordero, G., Kawamura, R., Sverzhinsky, A., Sarker, M., Roy, S., Malo, C., Pascal, J.M., Marko, J.F., and D'Amours, D. (2020). The smc5/6 core complex is a structure-specific DNA binding and compacting machine. Mol. Cell *80*, 1025–1038.e5. https://doi.org/10.1016/j.molcel.2020.11.011.

24. Huguet, J.M., Bizarro, C.V., Forns, N., Smith, S.B., Bustamante, C., and Ritort, F. (2010). Single-molecule derivation of salt dependent base-pair free energies in DNA. Proc. Natl. Acad. Sci. USA *107*, 15431–15436. https://doi.org/10.1073/pnas.1001454107.

25. Greene, E.C., Wind, S., Fazio, T., Gorman, J., and Visnapuu, M.-L. (2010). DNA Curtains for High-Throughput Single-Molecule Optical Imaging. Methods Enzymol *472*, 293–315. https://doi.org/10.1016/S0076-6879(10)72006-1.

26. Gutierrez-Escribano, P., Newton, M.D., Llauró, A., Huber, J., Tanasie, L., Davy, J., Aly, I., Aramayo, R., Montoya, A., Kramer, H., et al. (2019). A conserved ATP- and Scc2/4-dependent activity for cohesin in tethering DNA molecules. Sci. Adv. *5*, eaay6804–15. https://doi.org/10.1126/sciadv.aay6804.

27. Stigler, J., Çamdere, G.Ö., Koshland, D.E., and Greene, E.C. (2016). Single-molecule imaging reveals a collapsed conformational state for DNA-bound cohesin. Cell Rep. *15*, 988–998. https://doi.org/10.1016/j.celrep.2016.04.003.

28. Benham, C.J. (1992). Energetics of the strand separation transition in superhelical DNA. J. Mol. Biol. *225*, 835–847. https://doi.org/10.1016/0022-2836(92)90404-8.

29. SantaLucia, J. (1998). A unified view of polymer, dumbbell, and oligonucleotide DNA nearest-neighbor thermodynamics. Proc. Natl. Acad. Sci. USA *95*, 1460–1465. https://doi.org/10.1073/pnas.95.4.1460.

30. Terakawa, T., Bisht, S., Eeftens, J.M., Dekker, C., Haering, C.H., and Greene, E.C. (2017). The condensin complex is a mechanochemical motor that translocates along DNA. Science *358*, 672–676. https://doi.org/10.1126/science.aan6516.

31. Davidson, I.F., Goetz, D., Zaczek, M.P., Molodtsov, M.I., Huis in 't Veld, P.J., Weissmann, F., Litos, G., Cisneros, D.A., Ocampo-Hafalla, M., Ladurner, R., et al. (2016). Rapid movement and transcriptional re-localization of human cohesin on DNA. EMBO J. *35*, 2671–2685. https://doi.org/10.15252/embj.201695402.

32. Kanke, M., Tahara, E., Huis in't Veld, P.J., and Nishiyama, T. (2016). Cohesin acetylation and Wapl-Pds5 oppositely regulate translocation of cohesin along DNA. EMBO J. *35*, 2686–2698. https://doi.org/10.15252/embj.201695756.

33. Murayama, Y., and Uhlmann, F. (2014). Biochemical reconstitution of topological DNA binding by the cohesin ring. Nature *505*, 367–371. https://doi.org/10.1038/nature12867.

34. Gibb, B., Silverstein, T.D., Finkelstein, I.J., and Greene, E.C. (2012). Single-stranded DNA curtains for real-time single-molecule visualization of protein-nucleic acid interactions. Anal. Chem. *84*, 7607–7612. https://doi.org/10.1021/ac302117z.

35. Wold, M.S. (1997). Replication protein A: a heterotrimeric, single-stranded DNA-binding protein required for eukaryotic DNA metabolism. Annu. Rev. Biochem. *66*, 61–92. https://doi.org/10.1146/annurev.biochem.66.1.61.

36. Chen, J., Le, S., Basu, A., Chazin, W.J., and Yan, J. (2015). Mechanochemical regulations of RPA's binding to ssDNA. Sci. Rep. *5*, 9296–9298. https://doi.org/10.1038/srep09296.

37. Alani, E., Thresher, R., Griffith, J.D., and Kolodner, R.D. (1992). Characterization of DNA-binding and strand-exchange stimulation properties of y-RPA, a yeast single-strand-DNA-binding protein. J. Mol. Biol. *227*, 54–71. https://doi.org/10.1016/0022-2836(92)90681-9.

38. Kim, H., and Loparo, J.J. (2016). Multistep assembly of DNA condensation clusters by SMC. Nat. Commun. *7*, 10200–10212. https://doi.org/10.1038/ncomms10200.

39. Countryman, P., Fan, Y., Gorthi, A., Pan, H., Strickland, J., Kaur, P., Wang, X., Lin, J., Lei, X., White, C., et al. (2018). Cohesin SA2 is a sequence-independent DNA-binding protein that recognizes DNA replication and repair intermediates. J. Biol. Chem. *293*, 1054–1069. https://doi.org/10.1074/jbc.M117.806406.

40. Gorman, J., Wang, F., Redding, S., Plys, A.J., Fazio, T., Wind, S., Alani, E.E., and Greene, E.C. (2012). Single-molecule imaging reveals target-search mechanisms during DNA mismatch repair. Proc. Natl. Acad. Sci. USA *109*, E3074–E3083. https://doi.org/10.1073/pnas.1211364109.

41. Etheridge, T.J., Villahermosa, D., Campillo-Funollet, E., Herbert, A.D., Irmisch, A., Watson, A.T., Dang, H.Q., Osborne, M.A., Oliver, A.W., Carr,

A.M., and Murray, J.M. (2021). Live-cell single-molecule tracking highlights requirements for stable smc5/6 chromatin association in vivo. Elife *10*, 685799–e68619. https://doi.org/10.7554/ELIFE.68579.

42. Alt, A., Dang, H.Q., Wells, O.S., Polo, L.M., Smith, M.A., McGregor, G.A., Welte, T., Lehmann, A.R., Pearl, L.H., Murray, J.M., and Oliver, A.W. (2017). Specialized interfaces of Smc5/6 control hinge stability and DNA association. Nat. Commun. *8*, 14011–14014. https://doi.org/10.1038/ncomms14011.
43. Zabrady, K., Adamus, M., Vondrova, L., Liao, C., Skoupilova, H., Novakova, M., Jurcisinova, L., Alt, A., Oliver, A.W., Lehmann, A.R., and Palecek, J.J. (2016). Chromatin association of the SMC5/6 complex is dependent on binding of its NSE3 subunit to DNA. Nucleic Acids Res. *44*, 1064–1079. https://doi.org/10.1093/nar/gkv1021.
44. Taschner, M., Basquin, J., Steigenberger, B., Schäfer, I.B., Soh, Y.M., Basquin, C., Lorentzen, E., Räschle, M., Scheltema, R.A., and Gruber, S. (2021). Nse5/6 inhibits the Smc5/6 ATPase and modulates DNA substrate binding. EMBO J. *40*, e107807–e107823. https://doi.org/10.15252/embj.2021107807.
45. Roy, M.A., Siddiqui, N., and D'Amours, D. (2011). Dynamic and selective DNA-binding activity of Smc5, a core component of the Smc5-Smc6 complex. Cell Cycle *10*, 690–700. https://doi.org/10.4161/cc.10.4.14860.
46. Kanno, T., Berta, D.G., and Sjögren, C. (2015). The smc5/6 complex is an ATP-dependent intermolecular DNA linker. Cell Rep. *12*, 1471–1482. https://doi.org/10.1016/j.celrep.2015.07.048.
47. Kissling, V., Reginato, G., Bianco, E., Kasaciunaite, K., Tilma, J., Cereghetti, G., Lee, S.S., Guérois, R., Seidel, R., Cejka, P., et al. (2021). Mre11-Rad50 oligomerization promotes DNA double-strand break repair. Nat. Commun. *13*, 2374. https://doi.org/10.1038/s41467-022-29841-0.
48. Reuter, M., Zelensky, A., Smal, I., Meijering, E., van Cappellen, W.A., de Gruiter, H.M., van Belle, G.J., van Royen, M.E., Houtsmuller, A.B., Essers, J., et al. (2014). BRCA2 diffuses as oligomeric clusters with RAD51 and changes mobility after DNA damage in live cells. J. Cell Biol. *207*, 599–613. https://doi.org/10.1083/jcb.201405014.
49. Keenholtz, R.A., Dhanaraman, T., Palou, R., Yu, J., D'amours, D., and Marko, J.F. (2017). Oligomerization and ATP stimulate condensin-mediated DNA compaction. Sci. Rep. *7*, 14279–14313. https://doi.org/10.1038/s41598-017-14701-5.
50. Kim, Y., Shi, Z., Zhang, H., Finkelstein, I.J., and Yu, H. (2019). Human cohesin compacts DNA by loop extrusion. Science *366*, 1345–1349. https://doi.org/10.1126/science.aaz4475.
51. Badrinarayanan, A., Reyes-Lamothe, R., Uphoff, S., Leake, M.C., and Sherratt, D.J. (2012). In vivo architecture and action of bacterial structural maintenance of chromosome proteins. Science *80*, 528–531. https://doi.org/10.1126/science.1227126.
52. Varejão, N., Ibars, E., Lascorz, J., Colomina, N., Torres-Rosell, J., and Reverter, D. (2018). DNA activates the Nse2/Mms21 SUMO E3 ligase in the Smc5/6 complex. EMBO J. *37*, e98306. https://doi.org/10.15252/embj.201798306.
53. Whalen, J.M., Dhingra, N., Wei, L., Zhao, X., and Freudenreich, C.H. (2020). Relocation of collapsed forks to the nuclear pore complex depends on sumoylation of DNA repair proteins and permits Rad51 association. Cell Rep. *31*, 107635. https://doi.org/10.1016/j.celrep.2020.107635.
54. Miné-Hattab, J., Heltberg, M., Villemeur, M., Guedj, C., Mora, T., Walczak, A.M., Dahan, M., and Taddei, A. (2021). Single molecule microscopy reveals key physical features of repair foci in living cells. Elife *10*, 605777–e60656. https://doi.org/10.7554/eLife.60577.
55. Oshidari, R., Huang, R., Medghalchi, M., Tse, E.Y.W., Ashgriz, N., Lee, H.O., Wyatt, H., and Mekhail, K. (2020). DNA repair by Rad52 liquid droplets. Nat. Commun. *11*, 695. https://doi.org/10.1038/s41467-020-14546-z.
56. Claeys Bouuaert, C., Pu, S., Wang, J., Oger, C., Daccache, D., Xie, W., Patel, D.J., and Keeney, S. (2021). DNA-driven condensation assembles the meiotic DNA break machinery. Nature *592*, 144–149. https://doi.org/10.1038/s41586-021-03374-w.
57. Murayama, Y., Samora, C.P., Kurokawa, Y., Iwasaki, H., and Uhlmann, F. (2018). Establishment of DNA-DNA interactions by the cohesin ring. Cell *172*, 465–477.e15. https://doi.org/10.1016/j.cell.2017.12.021.
58. Gibb, B., Ye, L.F., Kwon, Y., Niu, H., Sung, P., and Greene, E.C. (2014). Protein dynamics during presynaptic-complex assembly on individual single-stranded DNA molecules. Nat. Struct. Mol. Biol. *21*, 893–900. https://doi.org/10.1038/nsmb.2886.
59. Vestergaard, C.L., Blainey, P.C., and Flyvbjerg, H. (2014). Optimal estimation of diffusion coefficients from single-particle trajectories. Phys. Rev. E Stat. Nonlin. Soft Matter Phys. *89*, 022726. https://doi.org/10.1103/PhysRevE.89.022726.
60. Kalafut, B., and Visscher, K. (2008). An objective, model-independent method for detection of non-uniform steps in noisy signals. Comput. Phys. Commun. *179*, 716–723. https://doi.org/10.1016/j.cpc.2008.06.008.
61. Candelli, A., Hoekstra, T.P., Farge, G., Gross, P., Peterman, E.J.G., and Wuite, G.J.L. (2013). A toolbox for generating single-stranded DNA in optical tweezers experiments. Biopolymers *99*, 611–620. https://doi.org/10.1002/bip.22225.

CellPress
OPEN ACCESS

STAR★METHODS

KEY RESOURCES TABLE

REAGENT or RESOURCE	SOURCE	IDENTIFIER
Antibodies		
Rat monoclonal anti-HA High Affinity (3F10)	Sigma-Aldrich	Cat#11867423001; RRID: AB_390918
Bacterial and virus strains		
Rosetta™(DE3)pLysS *E.coli*	Sigma-Aldrich	Cat#70956
Rosetta™(DE3) *E.coli*	Sigma-Aldrich	Cat#70954
Chemicals, peptides, and recombinant proteins		
DTT	Carl Roth	Cat#6908.4
β-mercaptoethanol	Fisher Scientific	Cat#10367100
ATP	Sigma-Aldrich	Cat#A26209-5G
Desthiobiotin	Fisher Scientific	Cat#12763064
SimplyBlue™ SafeStain	Fisher Scientific	Cat#LC6065
cOmplete, mini, EDTA-free protease-inhibitor mix	Sigma-Aldrich	Cat#4693159001
Pierce™ Universal Nuclease for Cell Lysis	Thermo Fisher	Cat#88702
d-biotin	Fisher Scientific	Cat#10746064
Biotin-14-dCTP	Jena Bioscience	Cat#NU-956-BIO14-S
Biotin-14-dATP	Jena Bioscience	Cat#NU-835-BIO14-S
BSA	Sigma-Aldrich	Cat#A7030-10G
Critical commercial assays		
StrepTrap HP	GE Healthcare	Cat#29-0486-53
HiTrap Heparin	GE Healthcare	Cat#17-0407-01
Superose 6 Increase 10/300 GL	GE Healthcare	Cat#29-0915-96
HisTrap HP	GE Healthcare	Cat#17-52348-01
HiTrap CaptoQ	GE Healthcare	Cat#11-0013-02
SiteClick Qdot 705 Antibody Labeling Kit	Fisher Scientific	Cat#15198735
SiteClick Qdot 605 Antibody Labeling Kit	Fisher Scientific	Cat#15136585
Experimental models: Organisms/strains		
Yeast strain: *MATa leu2-3,112 trp1-1 can1-100 ura3-1 ade2-1 his3-11,15 Lys2::pGAL1-GAL4::LYS2 pep4::HIS3 bar1::hisG*	Gutierrez-Escribano et al.[22]	N/A
Yeast strain: *MATa leu2-3,112 trp1-1 can1-100 ura3-1 ade2-1 his3-11,15 Lys2::pGAL1-GAL4::LYS2 pep4::HIS3 bar1::hisG [pRS424-GAL-SMC6-3xStrepII-SMC5-NSE4-His-3HA] [pRS426-GAL-NSE1-NSE3-NSE6-NSE2-NSE5]*	Gutierrez-Escribano et al.[22]	N/A
Oligonucleotides		
5′ Bioligo oligo 5′—BIOTEG—TTT TTT TTT TTT TTT TTT TTT TTT TTT TTT GTA AAA CGA CGG CCA GT	Metabion	N/A
ssDNA linker oligo 5′GGG CGG CGA CCT GGA CAG CTA GTG GCA CAA	Metabion	N/A
ssDNA-5′bio oligo 5′BIO-T(bio-dT)C TAT TCC ACT TCA ACT TTG TGC CAC TAG CTG TCC	Metabion	N/A

(*Continued on next page*)

Continued

REAGENT or RESOURCE	SOURCE	IDENTIFIER
capture01 oligo 5‘ATATATCTGACGCGTGCCTGG AGACTGGGGAGTAATCCTCTTG GCGGTT-Alexa594-AAA	IDT	N/A
capture02 oligo 5’TTTAACCGCCAAGAGGATTACTC CCCAGTCTCCAGGCACGCGTCAG ATATATACATCCTGT	Metabion	N/A
Recombinant DNA		
pRS424-GAL-SMC6-3xStrepII-SMC5-NSE4-His-3HA	Gutierrez-Escribano et al.[22]	N/A
pRS426-GAL-NSE1-NSE3-NSE6-NSE2-NSE5	Gutierrez-Escribano et al.[22]	N/A
p11d scRPA-mCherry-His	Gibb et al.[58]	N/A
pTXB3-φ29 DNA polymerase	Gibb et al.[34]	N/A
Software and algorithms		
Igor Pro 8	Wavemetrics	N/A

RESOURCE AVAILABILITY

Lead contact

Further information and requests for resources and reagents should be directed to and will be fulfilled by the lead contact, Johannes Stigler (stigler@genzentrum.lmu.de).

Materials availability

This study did not generate new, unique reagents.

Data and code availability statements

- Unprocessed data reported in this paper will be shared by the lead contact upon request.
- This paper does not report original code.
- Any additional information required to reanalyze the data reported in this paper is available from the lead contact upon request.

EXPERIMENTAL MODEL AND SUBJECT DETAILS

Yeast strains

The Smc5/6 holocomplex was expressed inW303 background *Saccharomyces cerevisiae* strain.

The strain used in this work is:

MATa leu2-3,112 trp1-1 can1-100 ura3-1 ade2-1 his3-11,15 Lys2::pGAL1-GAL4::LYS2 pep4::HIS3 bar1::hisG [pRS424-GAL-SMC6-3xStrepII-SMC5-NSE4-His-3HA] [pRS426-GAL-NSE1-NSE3-NSE6-NSE2-NSE5]

Yeast cells were grown at 30°C in selective drop-out media (-Trp, -Ura) supplemented with 2 % raffinose, 0.1 % glucose.

METHOD DETAILS

Plasmids

Plasmid for expression of Smc5/6 holocomplex:

GC1204 (pRS426-*GAL7promoter*-NSE1-NSE3-*GAL1-10promoter*-NSE6-NSE2-*GAL1-10promoter*-NSE5 URA3], GC1198 [pRS424-*GAL7promoter-SMC6*-3xStrepII *SMC5-GAL1-10promoter-NSE4*-8xHis-3xHA TRP1]

Expression and purification of Smc5/6 holocomplex

Expression plasmids used for the Smc5/6 holocomplex were described in a previous study.[22] Proteins were expressed and purified similar to a published protocol.[22] After transformation, yeast cells containing both plasmids were grown at 30°C in selective drop-out media supplemented with 2% raffinose, 0.1% glucose to an OD600 of 0.8–1.0. After expression was initiated by addition of 2% galactose, the cells were grown for further 16 h at 20°C and harvested by centrifugation (30 min, 4000 rpm, 4°C). Pellets were washed with PBS, resuspended in 2/3 volume 200 mM NaCl-Buffer A (25 mM Hepes pH 7.5, 5% glycerol, 5 mM β-mercaptoethanol)

containing cOmplete EDTA-free protease-inhibitor mix (Roche), frozen in liquid nitrogen and stored overnight at −80°C. Cells were then lysed in a freezer mill (SPEX Sample Prep Freezer/Mill 6970 EFM) and thawed for 2 h on ice before adding 1/3 volume 200 mM NaCl-Buffer A containing Pierce™ Universal Nuclease for Cell Lysis (0.1 μL/mL). After 1 h of further incubation at 4°C, the lysate was centrifuged (1 h, 17000 rpm, 4°C), the supernatant was filtered through 0.22 μm filters (Millipore) and loaded on a 1 mL Strep Trap™ HP (GE Healthcare). Protein was eluted with Buffer B (5 mM desthiobiotin in 200 mM NaCl-Buffer A), the fractions of interest were pooled and sample salt content was adjusted to 150 mM NaCl. Protein sample was filtered (0.22 μM, Millipore) and loaded on a 5 mL HiTrap™ Heparin (GE Healthcare) pre-equilibrated with 150 mM NaCl-Buffer A. Protein was eluted from the column with a linear gradient of 15–100 % Buffer A. The eluted protein fractions were pooled, concentrated (Amicon 100 kDa), adjusted to 300 mM NaCl and loaded on a size-exclusion column (Superose 6 Increase 10/300 GL, GE Healthcare). Fractions containing the Smc5/6 holocomplex were pooled, concentrated (Amicon 100 kDa), flash frozen, and stored at −80°C. Integrity of Smc5/6 holocomplex was confirmed by SDS PAGE (8% acrylamide gels stained with SimplyBlue™ SafeStain, ThermoFisher) and mass spectrometry (Table S1).

mCherry-RPA expression and purification

A plasmid (p11d) for mCherry tagged *S.cerevisiae* Replication Protein A (RPA)[58] was kindly provided by Dr. Bryan Gibb. The protein was expressed in Rosetta™(DE3)pLysS cells and purified similarly to a published protocol.[34] In brief, 1 L of cell culture was grown at 37°C to an OD600 of 1.0. The temperature was reduced to 18°C and expression was induced with 0.3 mM of IPTG. Cells were grown for another 18 h, harvested (centrifugation, 4000 rpm, 4°C), washed with PBS, flash frozen with liquid nitrogen and stored at −80°C. The cells were then thawed and resuspended in Buffer C (50 mM NaKPO4, 150 mM NaCl, 10 mM imidazole, pH 7.4) supplemented with protease inhibitor and 1 mM PMSF and lysed by sonication on ice. The supernatant was filtered (0.22 μM, Millipore) and loaded on a 5 mL His Trap™ HP column (GE Healthcare). After a wash with Buffer D (50 mM NaKPO4, 150 mM NaCl, 20 mM imidazole, pH 7.4), the protein was eluted from the column with a gradient of 0–100% Buffer E (50 mM NaKPO4, 150 mM NaCl, 1 M imidazole, pH 7.4). Peak fractions were pooled, dialyzed against 1 L Buffer F (20 mM Tris pH 7.4, 40 mM NaCl, 1 mM DTT, 0.5 mM EDTA) and loaded on a 1 mL HiTRap™ CaptoQ™ (GE Healthcare) column preequilibrated with Buffer G (20 mM Tris pH 7.4, 1 mM DTT, 0.25 mM EDTA). Protein was eluted using a 4–30% linear gradient to Buffer H (20 mM Tris pH 7.4, 1 M NaCl,1 mM DTT, 0.25 mM EDTA). Fractions containing the protein of interest were pooled and dialyzed into 1 L Buffer I (20 mM Tris pH 7.4, 100 mM NaCl, 1 mM DTT, 0.1 mM EDTA), concentrated with PEG8000 and then dialyzed into Buffer J (20 mM Tris pH 7.4, 100 mM NaCl, 1 mM DTT, 50% glycerol).

Φ29 polymerase expression and purification

A plasmid containing 3xFLAG tagged Φ29 polymerase,[34] kindly provided by Dr. Bryan Gibb, was transformed into Rosetta (DE3) cells. Cells were grown in 1L of LB medium and protein expression was induced with 0.3 mM IPTG. Expression was carried out for further 16 h at 18°C. Cells were recovered (centrifugation, 4000 rpm, 4°C), resuspended in Buffer K (25 mM Tris−HCl pH 7.4, 500 mM NaCl, 5% glycerol, 5 mM imidazole, protease inhibitor) and lysed by sonication on ice. The lysate was then clarified (0.22 μM syringe filtered, Millipore) and loaded on a 5 mL His Trap™ HP column (GE Healthcare). After sample application, unbound proteins were washed from the column with Buffer L (25 mM Tris−HCl pH 7.4, 500 mM NaCl, 5% glycerol, 5 mM imidazole). Elution of the protein of interest was carried out with a 0–100% gradient of Buffer M (25 mM Tris−HCl pH 7.4, 500 mM NaCl, 5% glycerol, 500 mM imidazole). Protein fractions were pooled together and applied to a chitin resin (NEB). After a wash with Buffer N (25 mM Tris−HCl pH 7.4, 500 mM NaCl), the resin was incubated overnight at 4°C in 50 mM DTT in Buffer N allowing the recovery of the protein of interest. The protein sample was dialyzed into Buffer O (10 mM Tris-HCl, pH 7.4, 100 mM NaCl, 1 mM DTT, 0.1 mM EDTA, 50% glycerol).

DNA curtains setup

DNA curtain experiments were carried out as described previously[25] on a prism-type TIRF microscope (Nikon Eclipse Ti2), equipped with two illumination lasers (488 nm and 561 nm, Coherent OBIS), an electron multiplying charged coupled camera (iXon Life, Andor) and a syringe-pump-driven microfluidics system supplying the sample chamber. Custom made flow cells were assembled from silica-fused slides grafted with chromium barriers produced via E-beam lithography and cover slips with double sided tape. Videos were recorded in NIS Elements (Nikon) at 100, 200 or 500 ms resolution and were analyzed in Igor Pro 8 (Wavemetrics) using custom written code.

Experiments were carried out in Buffer R (40 mM Tris-HCl pH 7.5, 2 mM $MgCl_2$, 1 mM DTT, 1 mg/mL BSA) supplemented with an oxygen scavenger (glucose oxidase/catalase, 0.8% glucose) unless stated otherwise. For dsDNA visualization 0.16 nM YOYO-1 was added to the buffer. Smc5/6 complexes were visualized by labeling them with quantum dots (SiteClick™ Qdot™ 705 Antibody Labeling Kit, ThermoFisher) conjugated to anti HA antibodies (Anti HA High Affinity, clone 3F10, Roche).

For dsDNA curtains, DNA molecules from λ bacteriophage (NEB), labeled with biotin and digoxigenin at the ends, were tethered to the lipid bilayer (DOPC, DOPE PEG, DOPE biotin) passivated surface via biotin-streptavidin interaction for free DNA end measurements (single-tethered curtains) and additionally via digoxigenin-anti digoxigenin antibody interaction for double-tethered curtains. DNA was flow-stretched over the Cr barriers to align in an array.

For ssDNA curtains, ssDNA substrate was generated as described previously[34] from an M13mp18 (NEB) template. Briefly, the ssDNA substrate was annealed with 5′Bi-oligo in annealing buffer (10 mM Tris-HCl, pH 8.0, 50 mM NaCl, 10 mM $MgCl_2$) for 5 min

at 95°C and slowly cooled down to room temperature. The biotinylated template was then used in a rolling circle reaction with in-house purified Φ29 polymerase (3.6 μL from 7.8 μM protein stock, 60 min reaction time, quenched with 75 mM EDTA) generating long stretches of linear ssDNA (up to 70k nucleotides). Before applying the ssDNA to the flow cell, RPA was transiently loaded to allow, through its unspecific adsorption at the downstream barriers, tethering of the ssDNA molecules. The other DNA end was anchored to the surface through biotin-streptavidin binding. Secondary structures as well as excess template were flushed out with a pulse of 6 M Urea.

DNA curtains experiments

dsDNA binding experiments

dsDNA binding measurements were carried out on single-tethered DNA curtains. Smc5/6 samples were prepared by incubating the protein with Qdot705 in a 1:2 ratio for 10 min on ice in Buffer R supplemented with 25 mM KCl and 25 mM NaCl. Prior to injection on DNA curtains, 100 μM biotin (used throughout all the experiments) and, when indicated, 1 mM ATP, were added to the protein sample. Protein was incubated on DNA curtains for 20 min without flow. Unbound protein was flushed out afterwards and bound protein could be identified by moving with the DNA upon flow application.

Lifetime measurements

For lifetime measurements, Qdot705-tagged Smc5/6, prepared as before and supplemented with different nucleotides (none, ADP, ATPγS, or ATP), was loaded on a double-tethered DNA curtain. After protein arrival, the flow was stopped and videos were recorded for additional 10 min. To decrease photo-induced DNA damage, the DNA was illuminated only every 10th frame with the 488 nm laser. Lifetimes were determined from manually analyzing kymograms. Survival plots were obtained using a Kaplan-Meier estimator and error bars were estimated from bootstrapping. Curves were then fitted to a double exponential model $S(t) = (1 - f)e^{-\frac{t}{\tau_s}} + fe^{-t/\tau_l}$, where τ_s and τ_l are the short and long lifetimes, respectively, and f is the fraction of long lifetimes.

Diffusion experiments

In the case of diffusion experiments, after protein sample application, the buffer was exchanged to R buffer containing 0, 100 or 250 mM NaCl and then the flow was stopped. Fluorescent protein complexes were tracked individually by selecting regions of interest (ROI) that contain single complexes. Within the regions, the position was determined by fitting a two-dimensional Gaussian. Localizations with large fitting uncertainties or low fluorescence amplitudes were discarded. Diffusion constants were then determined on a per-complex basis using a published covariance-based estimator.[59]

End-dependent dissociation

To determine the dissociation position of Smc5/6 on single-tethered DNA molecules, protein sample supplemented with 1 mM ATP was loaded onto DNA curtains and the protein binding positions were recorded on buffer-flow-stretched DNA. The running buffer was then exchanged to Buffer R supplemented with 250 mM NaCl while maintaining the flow. Protein binding and dissociation positions were determined from kymograms.

ssDNA binding experiments

Measurements were conducted on naked double-tethered ssDNA curtains with Smc5/6 protein sample prepared as in the case of dsDNA curtains. Protein was loaded on established curtains, videos were recorded and histograms of the binding positions Smc5/6 were generated.

Second-capture of dsDNA and ssDNA

Capture assays were conducted with unlabeled protein (in 50 mM NaCl supplemented R buffer, 1 mM ATP) loaded onto double-tethered DNA curtains. After a 5 min incubation, 16 nM AlexaFluor-594 end-labeled 61-mer oligonucleotide (capture01) were injected in the sample chamber. After flush-out of excess oligonucleotides and further incubation, Qdots were injected for protein visualization. The same setup was used for dsDNA capturing. Double-stranded DNA used in that case was generated from the ssDNA oligonucleotide used in the ssDNA capture assay through annealing with the complementary oligonucleotide (capture02). The annealing reaction was carried out for equimolar single-stranded oligonucleotides in annealing buffer (10 mM Tris pH 7.5, 50 mM NaCl, 1 mM EDTA). The reaction mixture was incubated for 5 min at 95°C and slowly cooled down to room temperature.

After recording videos, fluorescence trajectories $F(t)$ of captured oligonucleotides were analyzed using a modified step-finding algorithm based on work by Kalafut et al.,[60] where a constraint of constant step size (corresponding to the fluorescence f of a single fluorophore) was introduced. Step size histograms showed clear peaks for the photobleaching of one or multiple fluorophores, as well as the occasional reappearance of fluorescence due to fluorophore blinking (Figures S3C and S3E). The fluorescence of a single fluorophore was estimated from gaussian fits to these histograms. The number of captured oligonucleotides in a punctum was then determined from the initial fluorescence amplitude of a photobleaching trajectory as $F(0)/f$.

DNA compaction

For compaction experiments, 50 nM Smc5/6 (sample buffer: 40 mM Tris-HCl, pH 7.5, 50 mM NaCl, 4 mM $MgCl_2$, 1 mM DTT, 100 μM biotin) in presence of 2 mM ATP were loaded onto single-tethered curtains under different flow conditions. For no flow experiments,

protein was loaded under high flow (300 μL/min), followed by incubation in absence of flow for 15 min. For continuous flow experiments, protein was initially loaded under high flow (5—10 s) and then the flow rate was changed to low flow (10 or 20 μL/min) until most of the protein was loaded. Compaction was verified by applying high flow at the end of the measurements, followed by injection of 250 mM NaCl which allowed compacted DNA stretches to recover to full length. DNA initial and compacted lengths determined from kymograms allowed an estimation of compaction rates under the specified conditions.

Oligomerization

To determine the degree of Smc5/6 oligomerization, dual color labeling experiments were carried out on double-tethered curtains. Protein sample was prepared in 50 mM NaCl-Buffer R by incubating Smc5/6, with the same quantity of 2 different colors quantum dots (Qdot605 and Qdot705) which can be detected on two distinct camera channels. Buffer was exchanged to 250 mM NaCl for some experiments to induce diffusive motion.

The degree of oligomerization was determined from a statistical model. Given a cluster with N labelable units, the PMF of the numbers c, m of units to acquire cyan and magenta labels, respectively, is given by a trinomial distribution $f(c,m;N,p_c,p_m) = \frac{N!}{c!m!(N-c-m)!}p_c^c p_m^m(1-p_c-p_m)^{N-c-m}$, which we parameterize by the overall labeling efficiency $\kappa = p_c + p_m$ and the labeling asymmetry ratio $\gamma = p_c/p_m$, which accounts for differences between the Qdot labels.

From this, we determine the probability that a detected cluster of fixed size N has only cyan or only magenta labels

$$p_{cOnly} = \frac{1}{\alpha}\sum_{c=1}^{N} f(c,0;N,\kappa,\gamma),\ p_{mOnly} = \frac{1}{\alpha}\sum_{m=1}^{N} f(0,m;N,\kappa,\gamma),$$

where the normalization factor $\alpha = 1 - f(0,0;N,\kappa,\gamma)$ is the probability that the cluster carries a label at all. The probability of acquiring labels of both colors is then $p_{dual} = 1 - p_{cOnly} - p_{mOnly}$.

To account for the possibility that cluster sizes may not be equal, we introduce the distribution of cluster sizes $\phi(N;\mu)$, parameterized by the mean cluster size μ. For the general case of non-fixed cluster sizes, the probability that any given cluster carries only cyan or only magenta labels is then ($i \in \{cOnly, mOnly\}$)

$$p_i^{tot}(\mu) = \sum_{N=1}^{\infty} \phi(N;\mu) p_i.$$

The probability that a given cluster has two labels is $p_{dual}^{tot} = 1 - p_{cOnly} - p_{mOnly}$.

While the calculation can in principle be done for many distributions ϕ, we here considered two distribution models: (1) All clusters have the same size: $\phi(N;\mu) = 1\ (N = \mu)\ \mathit{and}\ 0\ (\mathit{otherwise})$. (2) Clusters have a stochastic distribution, modeled by a truncated Poissonian: $\phi(N;\mu) = \mu^N e^{-\mu}/(N!(1-e^{-\mu}))$.

The labeling asymmetry ratio γ was found by comparing the observed labeling ratio with the model-predicted one. In addition, γ can also be estimated from a maximum-likelihood approach. In either case, we find $\gamma = \sim 1.2$–1.4. Importantly, the shape of the likelihood function only weakly depends on γ (Figure S4G) and the conclusions are unaffected by asymmetric labeling.

Optical tweezers setup & measurement modes

Optical tweezers experiments were conducted on a dual-trap C-trap (LUMICKS) with microfluidics and confocal microscopy (488 nm, 561 nm and 638 nm lasers) capabilities. Experiments were recorded using Bluelake software provided by LUMICKS and data obtained was analyzed with custom written code in Igor Pro 8. Measurements were carried out in a five-channel flow cell, a mobile stage allowing seamless optical trapping in each of the 5 channels. Channel 1–3 separated by laminar flow were used for DNA trapping, while the remaining two orthogonal channels were used for protein loading and buffer exchange respectively. Streptavidin coated polystyrene beads (4.38 μm, SPHERO^{TM} Streptavidin Polystyrene, Spherotech) were trapped and subsequently moved to the DNA channel. Tethering was established through cyclic movement of the mobile trap towards and away from the fixed trap under flow. Experiments were carried out at room temperature in a buffer containing 50 mM Tris-HCl, pH 7.5, 50 mM NaCl, 4 mM $MgCl_2$, 1 mM DTT, 0.5 mg/mL BSA, supplemented with 1 mM ATP. Buffer salt concentration was exchanged as indicated to 150 or 250 mM NaCl. An oxygen scavenger system (glucose oxidase/catalase, 0.8% glucose) was added to the imaging channels and 100 μM biotin to the protein sample. For visualization of ssDNA tracts 16 nM mCherry-RPA (561 nm laser line) was used while 0.16 nM YOYO-1 (488 nm laser line) was used for dsDNA tracts when indicated. Protein was labeled, as described for DNA curtains, by incubating 5 nM Smc5/6 complex on ice with twice the amount of anti HA Qdot705. Fluorescence was recorded either as image scans or as line scans (kymograms) along the DNA molecule. For visualization only, each scan line was passed through a Richardson-Lucy deconvolution filter. All image quantification was performed on non-deconvolved raw image data.

λ-DNA dsDNA substrate with multiple biotins at both 5′ and 3’ of the same DNA strand was purchased from LUMICKS und used for preliminary measurements. Subsequent experiments were carried out using an in house triple end biotin tagged DNA. To generate this substrate, a similar strategy to a previously published one[61] for creating double end biotinylated λ-DNA was employed. In brief, we sequentially ligated two oligonucleotides to λ-DNA prior to using Klenow exo-polymerase (NEB) to fill in the ends with biotinylated nucleotides. Firstly, 10 μM phosphorylated oligo (ssDNA linker) was annealed and ligated into 14 nM phosphorylated linear DNA

(0.02 U/μL T4 Polynucleotide Kinase, 0.02 U/μL T4 ligase). Then a biotinylated oligo (ssDNA-linker-5′bio) was annealed to the overhang created in the previous step. The construct was then purified by ethanol precipitation, resuspended in T4 ligase buffer and further modified through a Klenow reaction with biotin-14-dCTP and biotin-14-dATP (Jena Bioscience). T4 ligase was then added and the final product was purified either through ethanol precipitation or size exclusion chromatography on a Sephacryl 300 column (GE Healthcare, running buffer 10 mM Tris-HCl pH 8.0, 150 mM NaCl, 1 mM EDTA).

Optical tweezers experiments

Binding preference to dsDNA/ssDNA or junctions

ssDNA-dsDNA hybrids were generated from triple end biotinylated λ-DNA dsDNA through force induced melting at high forces (ca 65 pN) in mCherry-RPA channel and moved into the protein channel where kymograms of protein binding were recorded. Complexes for which a co-localization of RPA and Qdot signal was observed were assigned to ssDNA tracts while proteins binding on the dark region were assumed to be bound to dsDNA. Binding events with a fluorescence peak of the protein within 300 nm (~diffraction limit) of that of mCherry-RPA were considered to be located at ssDNA-dsDNA junction. The log2-enrichment $\log_2 E$ was calculated as $\log_2 E = \log_2 x - \log_2 a$, where x is the fraction of complexes assigned to a respective class (dsDNA, ssDNA or junction) and a is the fraction of available substrate of that class. For observing the unpeeled ssDNA region, tethers were moved to the protein channel area, allowing flow perpendicular to the tether, and exposed to gentle flow.

Dynamics of Smc5/6-DNA interaction

Protein bound DNA tethers obtained as above were dragged to the buffer channel containing a higher concentration of salt (150/250 mM NaCl) to promote diffusion. Complexes were classified based on their diffusion behavior as static or mobile, both at low salt and high salt.

Smc5/6 interaction with naked ssDNA tracts

To check if RPA hinders Smc5/6 diffusion on ssDNA tracts, RPA was substituted with YOYO-1. Tethered DNA molecules maintained under low forces, were briefly visualized using the 488 nm laser, before being moved to the protein channel. Protein visualization was achieved with the 561 nm laser line to prevent tether breakage. After protein binding, the force was increased progressively to generate ssDNA regions.

Dynamics of Protein-DNA interaction during force manipulation

Tethers containing ss and ds DNA regions, obtained through force induced melting, were moved to the protein channel. After protein binding, tethers were moved to the RPA channel and exposed to cyclic variation in inter-traps distance. RPA covered ssDNA tracts present at low force were counted and the ratio to the initial number of ssDNA tracts at high force was determined for each of the following scenarios regarding the end flanking of the tract at low force: one, two or no Smc5/6 present.

Size of RPA tracts during force manipulation

The size of RPA tracts during force manipulation was determined from polymer models. First, the extension ξ, *i.e.* the end-to-end distance, of the tracts was determined by an edge detecting algorithm at each single time point (white lines in Figures S6B and S6C). As the extension of any polymer depends on the applied force F, it was converted into a force-invariant contour length L (Figure S6A) by solving the WLC interpolation equation $F(\xi, L, p) = \frac{k_B T}{p}\left(\frac{1}{4}\left(1 - \frac{\xi}{L}\right)^{-2} - \frac{1}{4} + \frac{\xi}{L}\right)$ for L. The persistence length of RPA-coated ssDNA was set to 1.3–1.5 nm.[36]

QUANTIFICATION AND STATISTICAL ANALYSIS

Statistical analyses were performed using Igor Pro8. Unless indicated otherwise, error bars represent 68% confidence intervals from bootstrapping. Statistical significance was determined by appropriate tests, as indicated in the corresponding figure legends. Asterisks indicate the significance level: ns: $p \geq 0.05$, *: $p < 0.05$, **: $p < 0.01$, ***: $p < 0.001$. n represents the number of molecules/events. Additional details are given in the figure legends and the main text.

Cell Reports, Volume *41*

Supplemental information

Stabilization of DNA fork junctions by Smc5/6 complexes revealed by single-molecule imaging

Nicoleta-Loredana Tanasie, Pilar Gutiérrez-Escribano, Sigrun Jaklin, Luis Aragon, and Johannes Stigler

Supplemental information

Supplemental figures

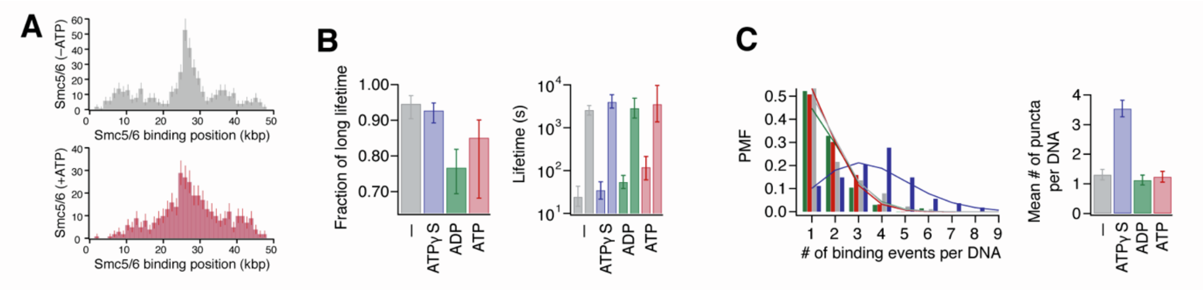

Figure S1. Nucleotides regulate the Smc5/6-DNA interaction, Related to **Fig. 1**. **(A)** Histograms of Smc5/6 binding locations in the absence of nucleotide (*n*=441, top) or in the presence of ATP (*n*= 366, bottom). **(B)** Fraction of long-lived population (left) and lifetimes of short-lived and long-lived populations (right) from double exponential fits to survival curves. Grey: no nucleotide, blue: ATPγS, green: ADP, red: ATP. **(C)** Left: distributions of the number Smc5/6 binding events per DNA, depending on nucleotide. Lines are fits to a Poissonian distribution model. Right: mean number of puncta per DNA depending on nucleotide. Color code as in (B). Data were collected from at least 50 DNA molecules per nucleotide condition. All error bars represent 68% CIs from bootstrapping.

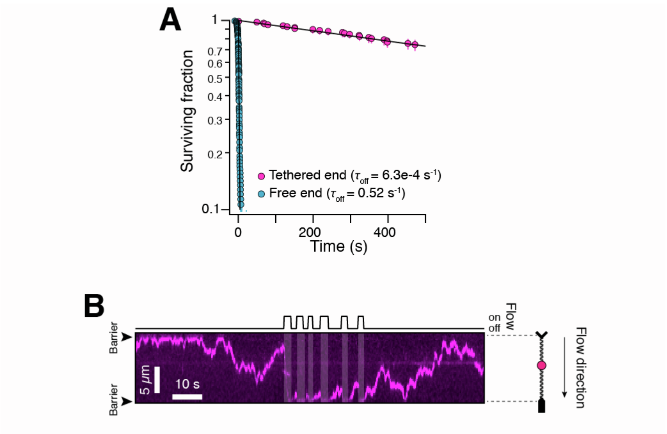

Figure S2. Smc5/6 interacts topologically with DNA, Related to **Fig. 2**. **(A)** Survival plots of moving complexes on DNA after an injection of 250 mM NaCl, on topologically unconstrained single-tethered DNA (*n*=180, cyan) and topologically constrained double-tethered DNA (*n*=97, magenta). Data were collected from at least 50 DNA molecules. **(B)** Smc5/6 movement on double-tethered DNA at 250 mM NaCl. Buffer flow pushes the complex toward the lower barrier.

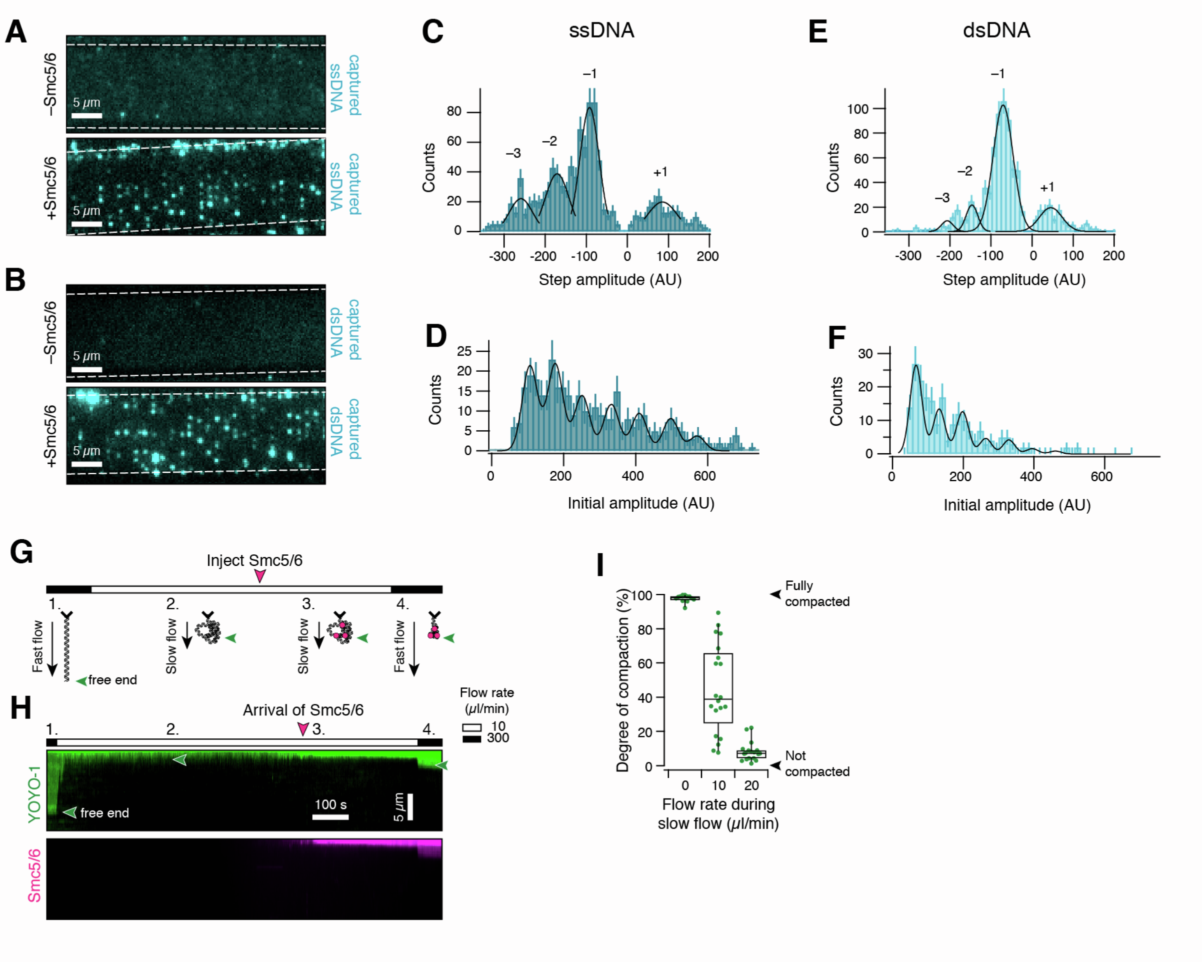

Figure S3. Smc5/6 can capture multiple ss or dsDNA oligos and compact dsDNA, Related to **Fig. 3**. **(A)** Wide-field images of dsDNA curtains with ssDNA-Alexa594 in solution. Top: without previous loading of Smc5/6 onto the curtain. Bottom: with previous loading of Smc5/6 onto the curtain. **(B)** Wide-field images of dsDNA curtains with dsDNA-Alexa594 in solution. Top: without previous loading of Smc5/6 onto the curtain. Bottom: with previous loading of Smc5/6 onto the curtain. **(C)** Detected step size histogram of photobleaching trajectories of captured ssDNA-Alexa594 oligos after flush-out. Black lines indicate the bleaching of a single fluorophore (–1), two fluorophores (–2), etc. Because the oligo was not completely removed from the flow cell, there are also occasional re-capture events (+1), seen by an increase in the fluorescence amplitude. **(D)** Histogram of the initial amplitude of captured ssDNA puncta after flush-out (n=387). Black lines represent a fit to the histogram with multiple gaussians corresponding to the fluorescence of one, two, etc. captured oligos. **(E,F)** see (C,D), but for the capture of dsDNA-Alexa594 (n=222). Histograms data were collected from at least 40 DNA molecules. Error bars are 68% confidence intervals. **(G)** Experimental profile of a flow-stretch compaction assay. **(H)** Representative kymogram of YOYO-1-stained DNA during compaction by Smc5/6 under buffer flow. Green arrowheads indicate the free end position. **(I)** The degree of compaction depends

on the buffer flow rate during the incubation phase. High flow, *i.e.* stretched DNA, prevents compaction. Data were collected from at least 12 DNA molecules per condition and are represented as box plots showing the median ± quartiles with whiskers indicating 9th and 91st percentiles.

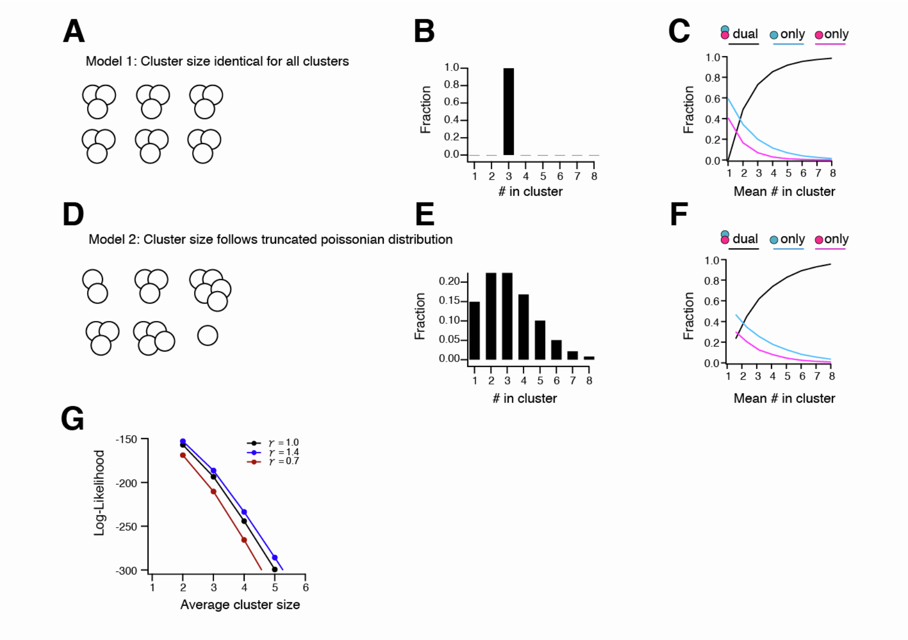

Figure S4. Models for determining the degree of oligomerization, Related to **Fig. 4**. **(A)** Cluster size model 1: All detected puncta have the same oligomeric size. **(B)** Distribution of oligomeric state according to model 1. **(C)** Prediction of the fraction of cyan-only-labeled, magenta-only-labeled and dual-color-labeled puncta according to model 1. **(D)** Cluster size model 2: The distribution of the number of molecules per punctum follows a truncated Poissonian distribution. **(E)** Assumed distribution according to model 2. **(F)** Predicted fraction of single and dual colored puncta according to model 2. **(G)** The likelihood function and its prediction of cluster size is largely unaffected by differences in the ratio γ of the labeling efficiency of cyan vs magenta labels.

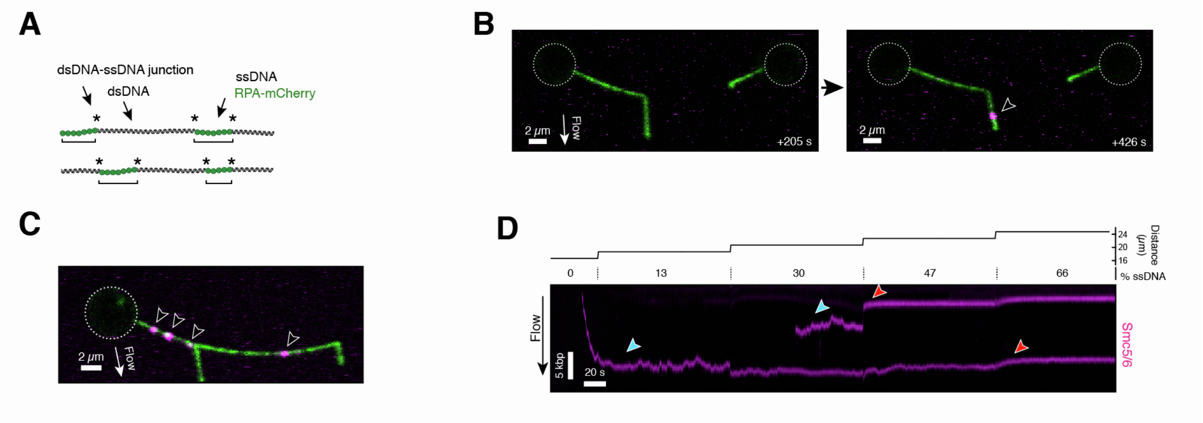

Figure S5. Smc5/6 interacts with ssDNA-dsDNA hybrids, Related to **Fig. 5**. **(A)** Schematic depiction of how the fractions of available dsDNA and ssDNA are determined (see **STAR Methods**). Asterisks denote junctions. **(B)** Perpendicular flow images of ssDNA/RPA-dsDNA hybrids. Green: RPA, magenta: Smc5/6. In this example, Smc5/6 binds directly to the unpeeled ssDNA strand (arrowhead). **(C)** Example where Smc5/6 complexes bind directly to the tethered ssDNA. **(D)** Kymogram of Smc5/6 movement on DNA during stepwise melting of the DNA. Blue arrows highlight mobile complexes. Higher trap distances, *i.e.* higher forces, result in local melting and increased exposure of ssDNA. Even in the absence of RPA, diffusing Smc5/6 complexes suddenly stop moving, presumably when they encounter ssDNA (red arrowheads).

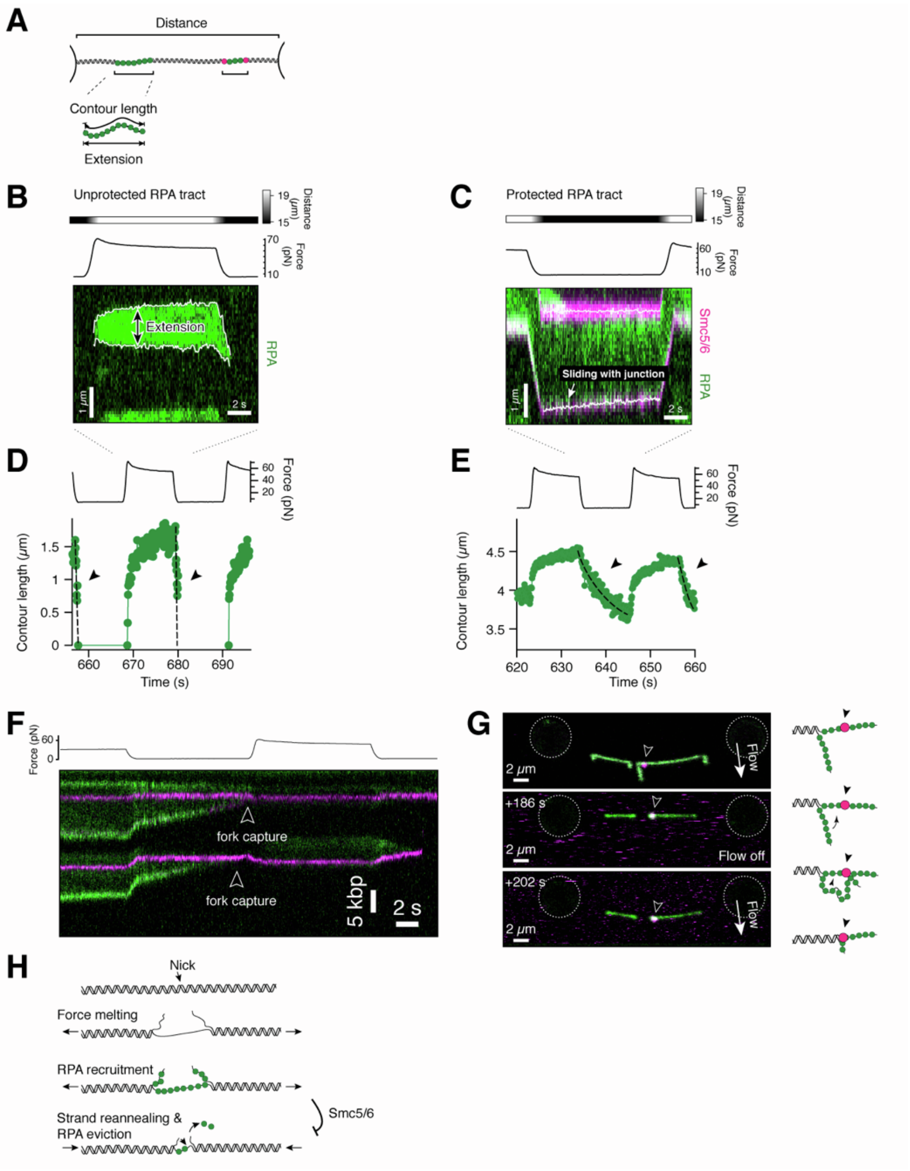

Figure S6. Smc5/6 associates with fork junctions, Related to **Fig. 6**. **(A)** The experimentally visible extensions of ssDNA tracts and dsDNA tracts are affected differently under force manipulation because ssDNA, even if it is RPA covered, has a much lower persistence length than dsDNA. The extension of tracts, which is directly experimentally accessible, is hence not a good reporter of the true tract length, *i.e.* the contour length. The shown schematic highlights this difference. **(B)** Kymogram of the extension of an unprotected RPA tract during force manipulation. White lines represent the RPA tract boundaries from tracking. **(C)** Kymogram of the extension of a protected RPA tract, *i.e.* with Smc5/6 bound to both tract junctions, during force manipulation. White lines represent the tract boundaries from tracking. **(D)** Contour length timeline of an unprotected RPA tract during force manipulation. The tract size rapidly returns to zero at low force (arrowheads). **(E)** Contour length timeline of a Smc5/6-protected RPA tract during force manipulation. The protected tract is kept open by Smc5/6. Nevertheless, even the protected tract shows slow dynamics due to sliding junctions (arrowheads). **(F)** Example kymogram showing that Smc5/6 bound to ssDNA associates with the ssDNA-dsDNA fork upon encounter (also see **Fig. 6E**). **(G)** Example video of

Smc5/6 (magenta) binding to the tethered ssDNA under perpendicular flow, followed by junction recruitment. At reduced force and in the absence of flow, the unpeeled ssDNA reanneals and the RPA to the left of the Smc5/6 complex is evicted. When the perpendicular flow is reinstated, Smc5/6 is at the junction and has possibly compacted part of the ssDNA. **(H)** Cartoon interpretation of the data in **Fig. 6**. An increase in force exposes ssDNA regions around nicks and lesions which become covered by RPA. In the absence of Smc5/6, reannealing of the displaced strand evicts RPA. This eviction is counteracted by the stabilization of ssDNA-dsDNA fork junctions by Smc5/6.

Supplemental tables

Table S1. Mass spectrometry verification of Smc5/6 holocomplexes, Related to **Fig. 1**. SM: Number of significant matches. SS: Number of significant sequences. emPAI: Exponentially Modified Protein Abundance Index

Accession	Score	Mass	SM	SS	emPAI	Description
**SMC6_YEAST**	27148	128568	784	73	9.24	Structural maintenance of chromosomes protein 6 OS=Saccharomyces cerevisiae (strain ATCC 204508 / S288c) OX=559292 GN=SMC6 PE=1 SV=1
**SMC5_YEAST**	10975	126481	408	75	9.97	Structural maintenance of chromosomes protein 5 OS=Saccharomyces cerevisiae (strain ATCC 204508 / S288c) OX=559292 GN=SMC5 PE=1 SV=1
**RLA0_YEAST**	104	33696	5	4	0.58	60S acidic ribosomal protein P0 OS=Saccharomyces cerevisiae (strain ATCC 204508 / S288c) OX=559292 GN=RPP0 PE=1 SV=2
**NSE5_YEAST**	15312	64784	511	22	2.71	Non-structural maintenance of chromosome element 5 OS=Saccharomyces cerevisiae (strain ATCC 204508 / S288c) OX=559292 GN=NSE5 PE=1 SV=1
**NSE4_YEAST**	2382	46150	93	19	3.88	Non-structural maintenance of chromosome element 4 OS=Saccharomyces cerevisiae (strain ATCC 204508 / S288c) OX=559292 GN=NSE4 PE=1 SV=1
**NSE3_YEAST**	6521	34002	265	20	9.77	Non-structural maintenance of chromosome element 3 OS=Saccharomyces cerevisiae (strain ATCC 204508 / S288c) OX=559292 GN=NSE3 PE=1 SV=1
**NSE2_YEAST**	1288	30857	53	9	2.06	E3 SUMO-protein ligase MMS21 OS=Saccharomyces cerevisiae (strain ATCC 204508 / S288c) OX=559292 GN=MMS21 PE=1 SV=2
**NSE1_YEAST**	6810	38890	235	11	1.98	Non-structural maintenance of chromosomes element 1 OS=Saccharomyces cerevisiae (strain ATCC 204508 / S288c) OX=559292 GN=NSE1 PE=1 SV=1
**KRE29_YEAST**	6637	54260	303	27	5.82	DNA repair protein KRE29 OS=Saccharomyces cerevisiae (strain ATCC 204508 / S288c) OX=559292 GN=KRE29 PE=1 SV=1
